# Clinical feature-related single-base substitution sequence signatures identified with an unsupervised machine learning approach

**DOI:** 10.1186/s12920-021-01144-1

**Published:** 2021-12-20

**Authors:** Hongchen Ji, Junjie Li, Qiong Zhang, Jingyue Yang, Juanli Duan, Xiaowen Wang, Ben Ma, Zhuochao Zhang, Wei Pan, Hongmei Zhang

**Affiliations:** 1 Department of Oncology, Xijing Hospital, Fourth Military Medical University, No. 127 West Changle Road, Xi’an, 710032 China; 2 Faculty of Hepatopancreatobiliary Surgery, Chinese PLA General Hospital, No. 28 Fuxing Road, Beijing, China; 3 Department of Emergency, Xijing Hospital, Fourth Military Medical University, No. 127 West Changle Road, Xi’an, China; 4 Department of Hepatoxbiliary Surgery, Xijing Hospital, Fourth Military Medical University, No. 127 West Changle Road, Xi’an, China

**Keywords:** Mutation sequence, Unsupervised learning, Cancer, Clinical feature, Prognosis

## Abstract

**Background:**

Mutation processes leave different signatures in genes. For single-base substitutions, previous studies have suggested that mutation signatures are not only reflected in mutation bases but also in neighboring bases. However, because of the lack of a method to identify features of long sequences next to mutation bases, the understanding of how flanking sequences influence mutation signatures is limited.

**Methods:**

We constructed a long short-term memory-self organizing map (LSTM-SOM) unsupervised neural network. By extracting mutated sequence features via LSTM and clustering similar features with the SOM, single-base substitutions in The Cancer Genome Atlas database were clustered according to both their mutation site and flanking sequences. The relationship between mutation sequence signatures and clinical features was then analyzed. Finally, we clustered patients into different classes according to the composition of the mutation sequence signatures by the K-means method and then studied the differences in clinical features and survival between classes.

**Results:**

Ten classes of mutant sequence signatures (mutation blots, MBs) were obtained from 2,141,527 single-base substitutions via LSTM-SOM machine learning approach. Different features in mutation bases and flanking sequences were revealed among MBs. MBs reflect both the site and pathological features of cancers. MBs were related to clinical features, including age, sex, and cancer stage. The class of an MB in a given gene was associated with survival. Finally, patients were clustered into 7 classes according to the MB composition. Significant differences in survival and clinical features were observed among different patient classes.

**Conclusions:**

We provided a method for analyzing the characteristics of mutant sequences. Result of this study showed that flanking sequences, together with mutation bases, shape the signatures of SBSs. MBs were shown related to clinical features and survival of cancer patients. Composition of MBs is a feasible predictive factor of clinical prognosis. Further study of the mechanism of MBs related to cancer characteristics is suggested.

**Supplementary Information:**

The online version contains supplementary material available at 10.1186/s12920-021-01144-1.

## Background

The stability of the cell genome is continually threatened by endogenous and exogenous factors that may lead to DNA damage [1, 2]. If not repaired properly, DNA damage may result in genetic mutations [3, 4]. The development of cancers involves a series of genetic mutations [5]. A number of internal and external factors underlying genetic mutations have been identified, such as smoking, alcohol consumption and mismatch repair deficiency [5, 6]. In some kinds of cancers, such as colon cancer [7] and breast cancer [8], there has been a great deal of research elucidating the relationship between genetic mutations and cancer-related processes.

Genetic mutations include single-base substitutions (SBSs), small insertions and deletions (indels), genome rearrangement and chromosome copy-number changes [9]. SBSs contribute the largest proportion of genetic mutations [9]. Mathematical methods have been used to decipher mutation signatures from somatic mutation catalogs [2, 9–16]. At present, large amounts of mutation data from cancer patients have been obtained and made available in relevant databases, such as the cancer genome atlas (TCGA) database [17]. In the context of increasing sample sizes, a number of mutation signatures that are correlated with certain mutation processes have been identified [18, 19]. The clustering methods for SBSs applied in some studies have included 1–2 bases next to mutated bases, and the results have suggested that bases next to the mutation site influence mutation signatures [2, 9]. However, the inclusion of adjacent genes in such analyses leads to an exponential increase in the number of possible classifications. Because of the lack of a highly efficient method to identify features of long sequences next to mutation bases, the understanding of how flanking sequences influence somatic mutation characteristics is limited.

The application of machine learning, especially neural networks, makes it possible to effectively mine information from large amounts of data [20]. A long short-term memory (LSTM) network is a special kind of recurrent neural network (RNN) [21]. Compared with a naive RNN, LSTM performs better in extracting features from long sequences, such as sentences [22, 23]. LSTM has been used to analyze DNA or RNA sequence information in some studies [24–26]. A self-organizing map (SOM) algorithm is an unsupervised clustering algorithm. The method of "competitive learning" can identify interconnections between samples and present their categories in a lower-dimensional form [27, 28]. The use of LSTM to extract the features of mutated sequences and the identification of similar features with the SOM algorithm provided an approach for analyzing the characteristics of mutated sequences and their relationship with cancer development. In this study, we established an LSTM-SOM unsupervised learning network to include long flanking sequences into the analysis of mutant sequence signatures. Via the LSTM-SOM method, we clustered the mutation sequences of SBS in the TCGA database into different classes (for a clear understanding, mutant sequence signatures clustered by the LSTM-SOM are referred to as mutation blots, MBs) and then analyzed the relationships among MBs, clinical features, and cancer patient survival.

## Methods

### Data sources

SBS data and clinical data of patients enrolled in this study were obtained from the TCGA database. First, the SBS information includes the sample barcode, chromosomal location, mutant allele, reference allele, Hugo gene symbol, etc. Clinical data, including age, sex, weight, cancer stage, and survival time or time to the last follow-up, were extracted according to the sample barcode. In the LSTM-SOM model, 100 flanking bases were included in the analysis, and the flanking sequence was obtained from the Genome Reference Consortium human genome build 38 (GRCh38) based on the mutation sites of SBSs in TCGA data.

### LSTM-SOM model building

In brief, our LSTM-SOM model works via a cycle of 3 steps: 1. extraction of the feature vector of the mutant sequence by LSTM; 2. clustering of feature vectors by the SOM, and feature vectors are updated at the same time to bring vectors with similar features closer together; and 3. use of the updated feature vectors for the labeling and training of the LSTM model (Additional file 1: Figure S1).

*Step 1*. *Obtaining feature vectors with LSTM* Mutant sequences are represented in the form of a matrix. A 1 × 2 vector is used to represent different bases (A: [0, 0]; T: [0, 1]; C: [1, 0]; G: [1, 1]; N:[− 1, − 1]). When placing the reference sequence in the corresponding position, mutated bases can be recorded as a 1 × 4 vector. When the flanking bases are included, a mutated sequence can be represented by an n × 4 matrix. For example, CATTG > CACTG can be expressed as follows:

$$\left[ {\begin{array}{*{20}c} 1 & 0 & 1 & 0 \\ 0 & 0 & 0 & 0 \\ 1 & 0 & 0 & 1 \\ 0 & 1 & 0 & 1 \\ 1 & 1 & 1 & 1 \\ \end{array} } \right]$$

RNNs have long been used in the analysis of sequence data. A naive RNN effectively analyzes short sequences. An LSTM network is based on the network structure of RNNs [25]. The LSTM approach introduces the mechanisms of "forgetting" and "memory". Thus, the capacity of the LSTM network to analyze long sequences is improved by controlling the long-term state [22]. As the "forgetting" mechanism of LSTM, the unit closer to the end of the sequences has a greater influence on the output of LSTM. In our study, LSTM was designed to read from both ends of the mutated sequence. In this way, the mutation site is placed at the ends of both sequences to reinforce its influence on the LSTM output.

We used the torch.nn package in PyTorch to construct a neural network. The LSTM procedure that we used consists of two hidden layers, each with 64 nodes. The data subsequently entered a full connection layer, and a 1 × 8 vector was finally output as the feature vector of a single mutated sequence.

*Step 2*. *Clustering with the SOM* The SOM consists of two kinds of layers: an input layer and a competition layer. The randomized units in the competition layer were trained to describe the distribution of units in the input layer via the mechanism of "competitive learning" [29]. In the SOM process of the LSTM-SOM model, the feature vector obtained from the LSTM process is used as the input. Units in the competition layer are adjusted continuously according to their distance to the input unit. For one input unit, the unit in the competition layer nearest to it is regarded as the "winning unit", which will move the maximal distance to the input unit (target), and for the other units, their travel distance to the target decreases with the increase in the distance to the winning unit. To avoid an excessive concentration of the results, we set a threshold value in the model. When the distance between the competition layer unit and the target is over the threshold value, the unit will move in the opposite direction to the target. In particular, not only will units in the competition layer be updated in our SOM model, but the input unit will also be updated in the opposite direction of the vector sum of the competition layer unit movement. Then, the updated input unit will be used as a label to train the LSTM model.

First, we obtained feature vectors of 100 samples from LSTM in one batch, and they were used as the input units of the SOM. The settings included 200 units in the SOM competition layer. For each input vector, the Euclidean distance between it (x) and each unit in the competition layer ($w_{j}$) was calculated as follows:

$$d_{j} \left( x \right) = \sqrt {\mathop \sum \limits_{i = 1}^{D} \left( {x_{i} - w_{{j_{i} }} } \right)^{2} }$$

The unit closest to x is recorded as $w_{min}$, and the distance between $w_{min}$ and each other competition layer unit is calculated as follows:

$$d_{j} \left( {w_{min} } \right) = \sqrt {\mathop \sum \limits_{i = 1}^{D} \left( {w_{{j_{i} }} - w_{{min_{i} }} } \right)^{2} }$$

A threshold of $S$ was set in the process of training. If $d_{j} \left( {w_{min} } \right) \le S$, $w_{j}$ will move in the direction of x; otherwise, $w_{j}$ will move in the opposite direction. The transportation distance decays with an increase in $d_{j} \left( {w_{min} } \right)$. The neighborhood function refers to the Gaussian function [29]:

$$D\left( {w_{j} } \right) = e^{{ - \frac{{d_{j} \left( {w_{min} } \right)^{2} }}{{2\pi \sigma^{2} }}}}$$

In the decay function, $\sigma$ is a constant that affects the amplitude of transportation distance decay. The update vector is as follows (where L is the learning rate of the SOM):

$${\Delta }\left( {w_{j} } \right) = \left\{ {\begin{array}{*{20}c} {L \times D\left( {w_{j} } \right) \times \left( {w_{j} - x} \right)} & {d_{j} \left( {w_{min} } \right) \le S} \\ { - L \times D\left( {w_{j} } \right) \times \left( {w_{j} - x} \right)} & {d_{j} (w_{min} ) > S} \\ \end{array} } \right.$$

When the distance between $w_{j}$ and the target $x$ is less than S, they will approach each other. Otherwise, they will pull away from each other. Due to the existence of the decay function, the influence of distant units on each other is very small, and no excessive dispersion of units was observed in training. To avoid overfitting, the units in the SOM competition layer are updated after each training batch of 100 samples. The samples in each batch are selected randomly from different cancers. To change the discrete status of the input vectors and cause similar input vectors to aggregate, the input units are updated in the opposite direction ($x$ is the input vector):

$$x\left( {new} \right) = x + \mathop \sum \limits_{j = 1}^{200} \Delta \left( {w_{j} } \right)$$

*Step 3*. *Training the LSTM model* The updated $x\left( {new} \right)$ is used as the label to train the LSTM network. In this way, the output feature vectors of LSTM with similar features can be gradually closed.

The above three steps are repeated until a clear, stable classification is obtained.

### Obtain the classification

During training, the units in the competition layer of the SOM were sorted according to the distance to $w_{min}$. S was set as the distance of unit rank 40 (5% of entire competition layer units) to $w_{min}$. After each iteration of SOM analysis, the updated input data were used as labels to train the LSTM model for 2 iterations. The LSTM learning rate was set as 0.001. The SOM learning rate was set as 0.005.

Two classes were obtained after one round of training. After 3 rounds of training, a total of 8 clustered classes were obtained. It was observed that there were 2 classes showing significantly larger sample sizes than the other classes. Therefore, an additional round of clustering was carried out in the 2 classes. Finally, we obtained 10 classes of mutated sequences.

### Analysis of clinical features

In the analysis of clinical features, measurement data were expressed as the mean ± standard deviation. In the analysis of differences between groups, an independent-samples T test (number of groups = 2) or analysis of variance (ANOVA) (number of groups > 2) was used. Enumeration data were expressed as count data, and chi-square analysis was used for difference testing. A sample was removed if the data of an item required for statistics were missing. *p* < 0.05 was considered to indicate a statistically significant difference. In the survival analysis, the log-rank test was used to analyze the difference in survival between different groups.

### Clustering of patients according to the MB composition

Patients were clustered according to their MB composition. In the clustering method according to the MB composition, each kind of MB was reflected as the percentage of the entire MB in one patient. The K-means method was used for clustering performed by the K-means method in the scikit-learn package. An "elbow method" was used to evaluate the K value (number of clustered groups) [30]. The K value evaluated in different cancers, and the entire sample was generally between 5–8. After comparing the clustering results, K = 7 was selected as the class number for K-means clustering.

### Code available

All mathematical methods were performed with Python. The code for the pretreatment of TCGA data and the construction, training and testing of the model is stored at https://github.com/FreudDolce/SBS_CLUSTER/. For clinical data analysis, patient clustering, survival analysis and drawing, the code is stored at https://github.com/FreudDolce/SATA/. All the code is open source and freely available.

## Results

### SBS clustering via the LSTM-SOM unsupervised machine learning approach

A total of 2,141,527 somatic SBS data points from 9596 patients were collected from the TCGA database. For each SBS sample, 100 flanking bases (50 bases at the 5′ end and 50 at the 3′ end) were included in the LSTM training data.

**Fig. 1:**
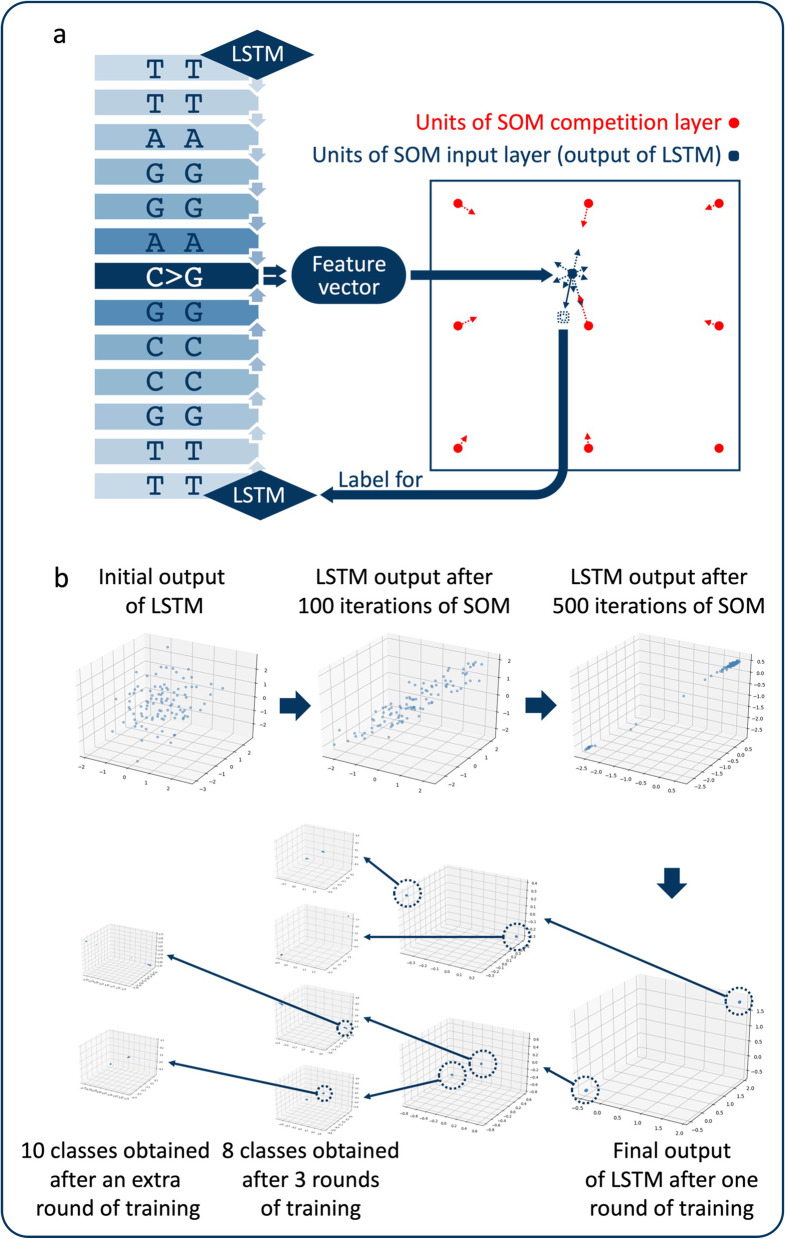
Training process of the LSTM-SOM model. **a** Sketch map of LSTM-SOM. **b** The clustering process. Two classifications were used for each training period. Ten classes of mutant sequences were obtained after 3 rounds and an extra round of training. Three of the eight dimensions in LSTM output vectors are shown in the space rectangular coordinate system

In brief, our LSTM-SOM model functions by extracting the features of mutant sequences via the LSTM network and then taking the generated feature vector as the input data for the SOM. Units in competitive layers of the SOM are then refreshed to edges closer to the distribution of the input data. After each iteration of the SOM in our LSTM-SOM model, not only will the units in the competitive layer of the SOM be refreshed, but the input data generated by LSTM will also be adjusted in the opposite direction (Fig. 1A). Then, the refreshed input data are used as the labels to train the LSTM model. The above steps were repeated until the LSTM outputs formed clear classifications.

One hundred samples from patients with different cancers were selected randomly in each training iteration. In the LSTM process, the influence of unit data on the LSTM output results decreased with increasing distance to the ending unit. The LSTM process was carried out on both sides of the mutation site in opposite directions. Thus, the mutation site was placed at the end of both sequences to expand its influence on LSTM output and to reflect the difference between the reference allele and mutant allele. Mutated sequences were clustered into 2 types after one stage of training. Thus, we obtained 8 classes of MBs after 3 stages of training. Then, an additional stage of training was performed for 2 classes of MB with a significantly larger number of samples and ultimately revealed 10 classes of MBs, recorded as MB 1–MB 10 (Fig. 1B).

### Features of mutation bases and flanking sequences in different MBs

**Fig. 2:**
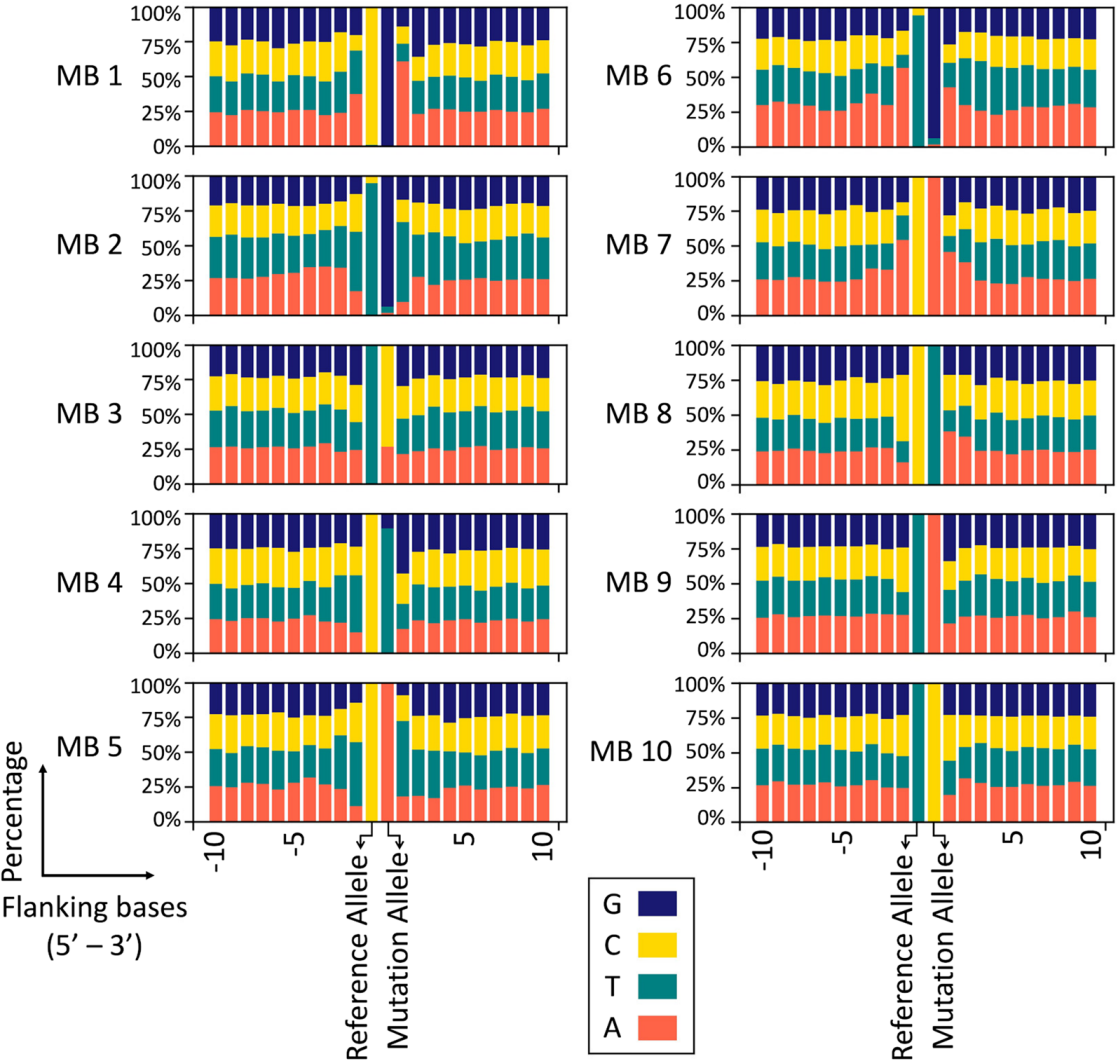
Mutation type and composition of flanking bases in different MBs. Each bar except for “Reference Allele” and “Mutation Allele” represents one flanking genetic locus. Bars on the left of “Reference Allele” represent bases on the 5’ end of the mutation site, and bars on the right of “Mutation Allele” represent bases on the 3’ end of the mutation site

Following the principle of complementary base pairing, 4 kinds of bases form 6 classes of base substitutions: C > A, C > G, C > T, T > A, T > C, and T > G, where base substitutions are represented by the pyrimidine residue of the base pair. Among the 10 classes of MBs clustered by the LSTM-SOM, 4 contained a single kind of mutation (MB 7: C > A; MB 8: C > T; MB 9: T > A; MB 10: T > C). The other 5 classes contained multiple types of mutations (MB 1: C > G, T > C, and T > G; MB 2: C > A, C > T, T > A and T > G; MB 3: T > A and T > C; MB 4: C > G and C > T; MB 5: C > A, C > G, T > C and T > G; MB 6: C > A, C > T, T > A and T > G) (Fig. 2, Additional file 2: Table S1).

The clustering results were strongly influenced by the flanking bases of the mutation site. For example, both MB 5 and MB 7 exhibited C > A mutations, and the flanking bases of MB 5 were dominated by T bases, but MB 7 was dominated by A bases. Differences in flanking bases could also be observed in other classes of MBs with similar mutation features, such as MB 2 and MB 6, MB 4 and MB 8 (Fig. 2). With an increase in the distance from the mutation site, the proportions of the four bases tended to become balanced. In the analysis of cancers with high incidence (lung, breast, prostate, colon, stomach, bladder, ovary, cervix uteri, liver, thyroid, skin and kidney cancers), the composition of the bases in the mutation site and the flanking sites of each MB basically followed that in the entire sample (Additional file 1: Figure S2).

### Study on MBs difference in cancers of multiple organ origin and pathologic types

**Fig. 3:**
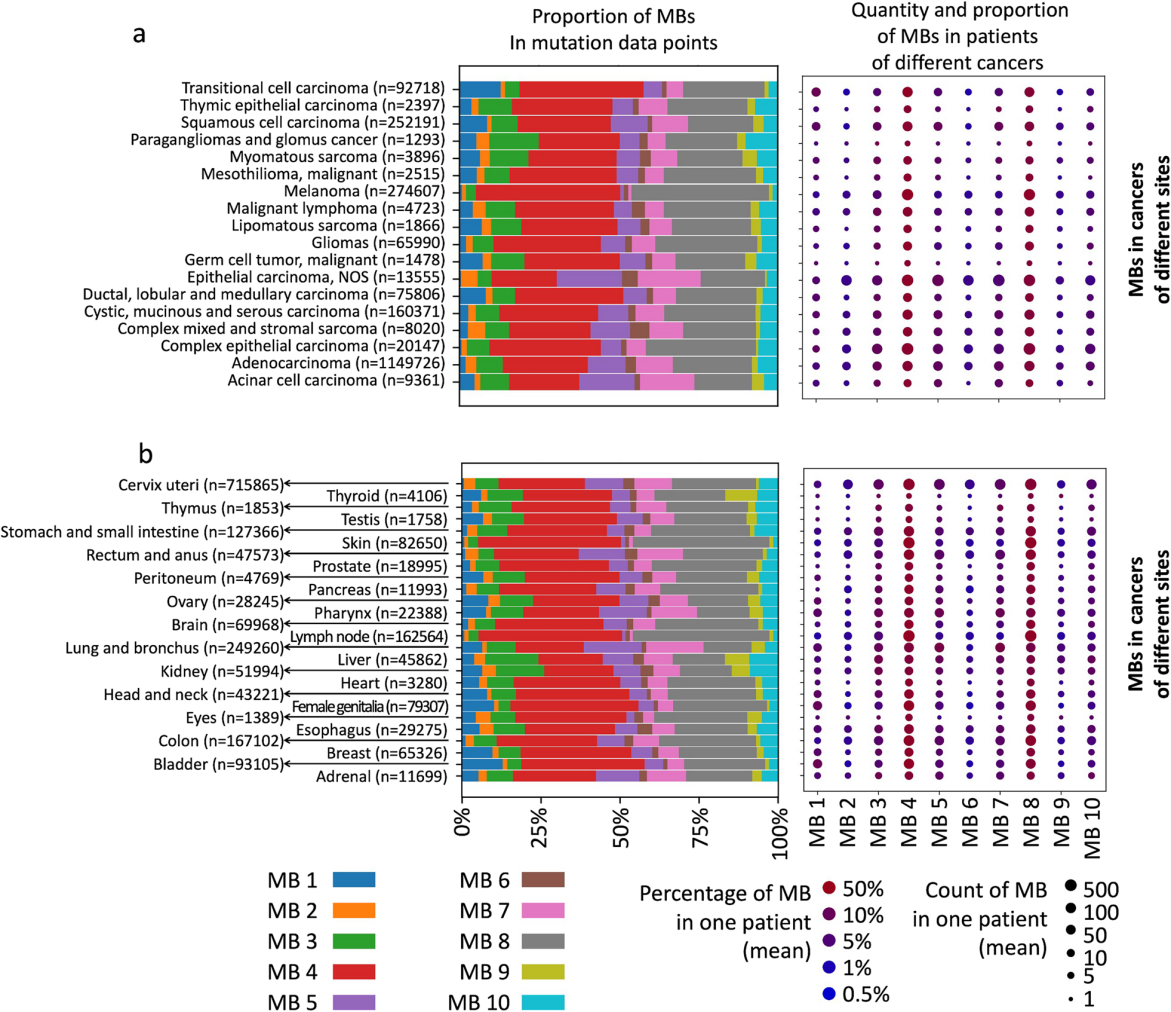
Quantity and proportion of MBs in different cancers. **a** MBs in cancers of multiple organ origin. **b** MBs in cancer of different pathologic types. The left subgraph shows the proportion of different MBs in all SBS mutation data points from different kinds of cancers. The right subgraph shows the quantity and proportion of different MBs in patients. Differences in quantity are reflected in the size of the point, and differences in proportion are reflected in the color of the point

Significant differences in the composition of MBs existed among cancers with different pathologies. Overall, MB 4, MB 5, MB 7 and MB 8 accounted for much greater percentages of the MBs than the other classes of MBs, especially MB 4 and MB 8 (Fig. 3A). Malignant mesenchymal tumors seemed to present a higher percentage of MB 2 and MB 6 than epithelial malignant tumors. Transitional cell carcinoma of the urinary tract showed a distinctly higher MB 1 incidence than other cancers. Cancers of germ cells and the glomus (paragangliomas) exhibited a high proportion of MB 10. An obvious feature of melanomas was the dominance of MB 4 and MB 8. This finding suggested that these classes of MBs may be correlated with ultraviolet light exposure.

The components of MBs varied in different cancers, and some cancers presented distinct features. The proportions of MBs in different cancers were influenced by the pathological type to some extent (Fig. 3B; Additional file 1: Figure S3). For example, cancers of the skin and lymph nodes showed extraordinarily high proportions of MB 4 and MB 8 but small proportions of other MBs. In both types of cancers, melanoma is the major pathologic type. Lung cancer presented high proportions of MB 5 and MB 7. Among the 2 major pathological types of lung cancer, adenocarcinoma (AC) exhibited much higher proportions of MB 5 and MB 7 than did squamous cell carcinoma (SCC). This was consistent with the MB composition in the two pathological types. However, for the same pathological type, differences in the MB composition could be observed in different cancers. For example, AC of the colon presented higher proportions of MB 4 and MB 8 than did AC of the lung. SCC of the lung exhibited more MB 5 and MB 7 than SCC in the head and neck (Additional file 1: Figure S4).

In most classes of MBs, the frequency of genes that were commonly mutated in malignant tumors, such as TTN, TP53, and MUC16, was relatively high. Distinct features existed in some classes of MB. The proportion of TP53 mutations was generally high, but it was relatively low in MB 7 and MB 10. Remarkably, BRAF was the most common mutated gene in MB 9. A higher proportion of PIK3CA mutations was observed in MB 8 and MB 10 than in the other classes of MBs (Additional file 2: Table S2). More distinct features could be observed when considering specific cancers. For example, in pancreatic cancer, MB 4 and MB 5 contained a higher frequency of KRAS mutations than did the other classes of MBs. In kidney cancer, the frequency of VHL mutations ranked high in MB 3, MB 5, MB 7 and MB 9. In skin and thyroid cancers, BRAF mutations were common in MB 9 but not in the other classes of MBs (Additional file 1: Figure S5).

### Survival analysis of patients with different MBs in the same mutation gene

**Fig. 4:**
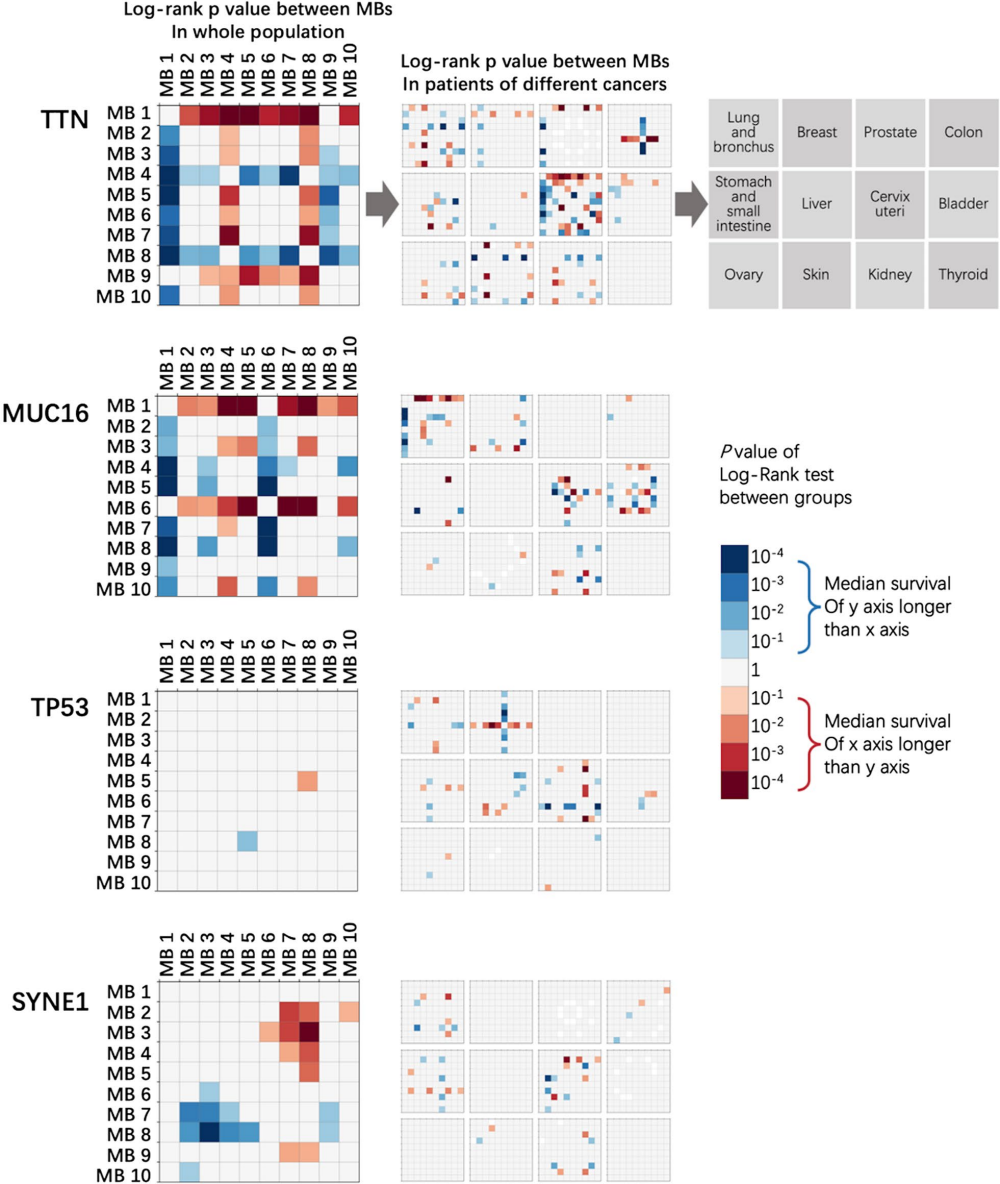
Relationship between patient survival and MB in genes with high mutation frequencies. The top 4 most frequently mutated genes are shown (other genes with high mutation frequencies are shown in Additional file 1: Fig. S5). For each gene, the left subgraph shows the p value of the log-rank test between groups in the whole population; and the right subgraph shows the p value of the log-rank test between groups of patients with different cancers with high incidence. Only p values less than 0.05 are shown in the heat map

In the mutated genes with a high frequency, the composition of MBs varied between different kinds of cancers. Such differences reflected the overall MB composition of each cancer (Additional file 1: Figure S6). To further study the influence of certain genes with different MBs on survival, we analyzed the survival of patients who exhibited mutations in genes with high mutation frequencies (TTN, MUC16, TP53, DNAH5, USH2A, PIK3CA, SYNE1, etc.). Patients were grouped according to the MB classification of specific genes. Patients carrying genes with MB 4 and MB 8 mutations usually showed better survival. In contrast, MB 1, 6, and 9 in a gene could predict worse survival (Fig. 4 and Additional file 1: Figures S7 and S8).

### Relationship between MBs and clinical features of cancer patients

**Fig. 5:**
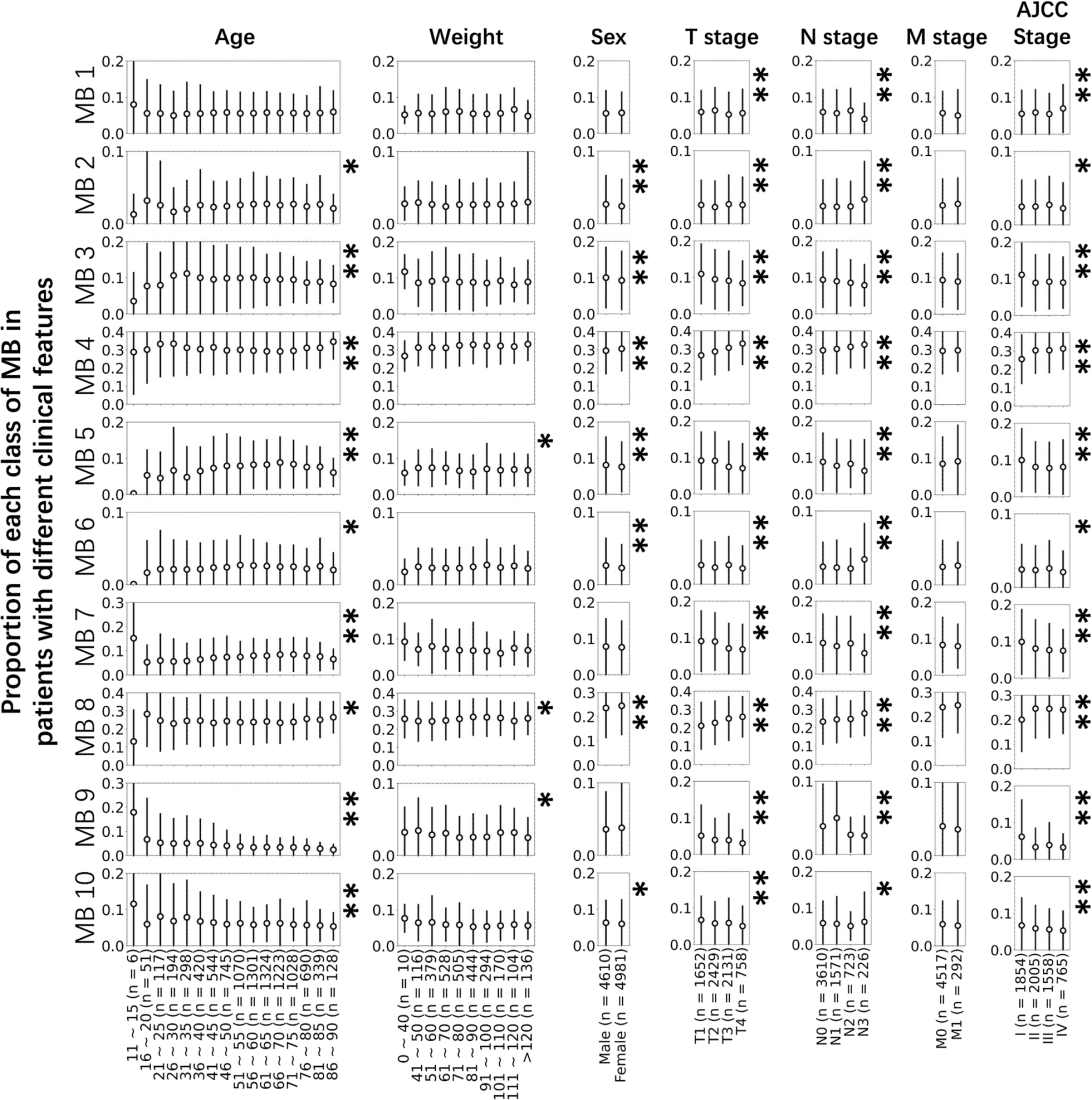
MBs in patients with different clinical features. *: *p* < 0.05 in the t test or ANOVA between groups; **p < 0.005 in the t test or ANOVA between groups. The proportion is shown as the mean ± standard deviation, and error bars represent standard deviation

Analysis was performed to determine the relationship between MBs and the clinical features of tumor patients, including their age, sex, weight, AJCC stage and TNM stage. The change in MBs showed a nonmonotonic trend with patient age. The percentages of MB 2, MB 5, and MB 7 in single patients increased with age within the first interval (< 70 for MB 2; < 75 for MB 5 and MB 7) but decreased when age exceeded the threshold. This trend was reversed for MB 4 and MB 8. An exception was observed for MB 9, whose proportion in single patients decreased monotonically with age. The proportion of MBs in a single patient generally varied between the sexs. Female patients were likely to show higher percentages of MB 2, MB 3, MB 5, MB 6 and MB 10, while male patients exhibited higher percentages of MB 4, MB 8 and MB 9. The difference was not significant in MB 1 and MB 7. No apparent rule regarding the relationship between the weight and MB composition of a patient was observed (Fig. 5 and Additional file 1: Figure S9).

Although the detailed methods of AJCC staging in different cancers are not the same, they generally follow similar principles [31]. Therefore, we merged the subdivisions of the stages in some cancers to analyze cancer stage. The proportions of MB 3, MB 7 and MB 9 showed a decreasing trend with increasing T and N stages. In contrast, MB 4 and MB 8 had a positive relationship with T and N stages. For some MBs, their relationship with cancer staging was complicated. MB 5 decreased with the progression of T and N stages, but M1 patients presented more MB 5 than M0 patients. MB 2 and MB 6 exhibited a remarkably high prevalence in N3 patients (Fig. 5 and Additional file 1: Figure S9).

In most cancers, the MB composition at different ages basically followed the pattern shown in the total samples. The proportion of MB 2 in most cancers was significantly higher in males than in females. Regarding cancer staging, T and M stages showed obvious tendencies in most kinds of cancers, and their trends were basically consistent with those for the total sample. Stomach cancer and colon cancer, in particular, showed opposite MB tendencies in T and N stages compared with the entire sample and with other cancers with high incidence (Additional file 1: Figures S10–S13).

### Composition of MBs in cancer patients is related to clinical prognosis

**Fig. 6:**
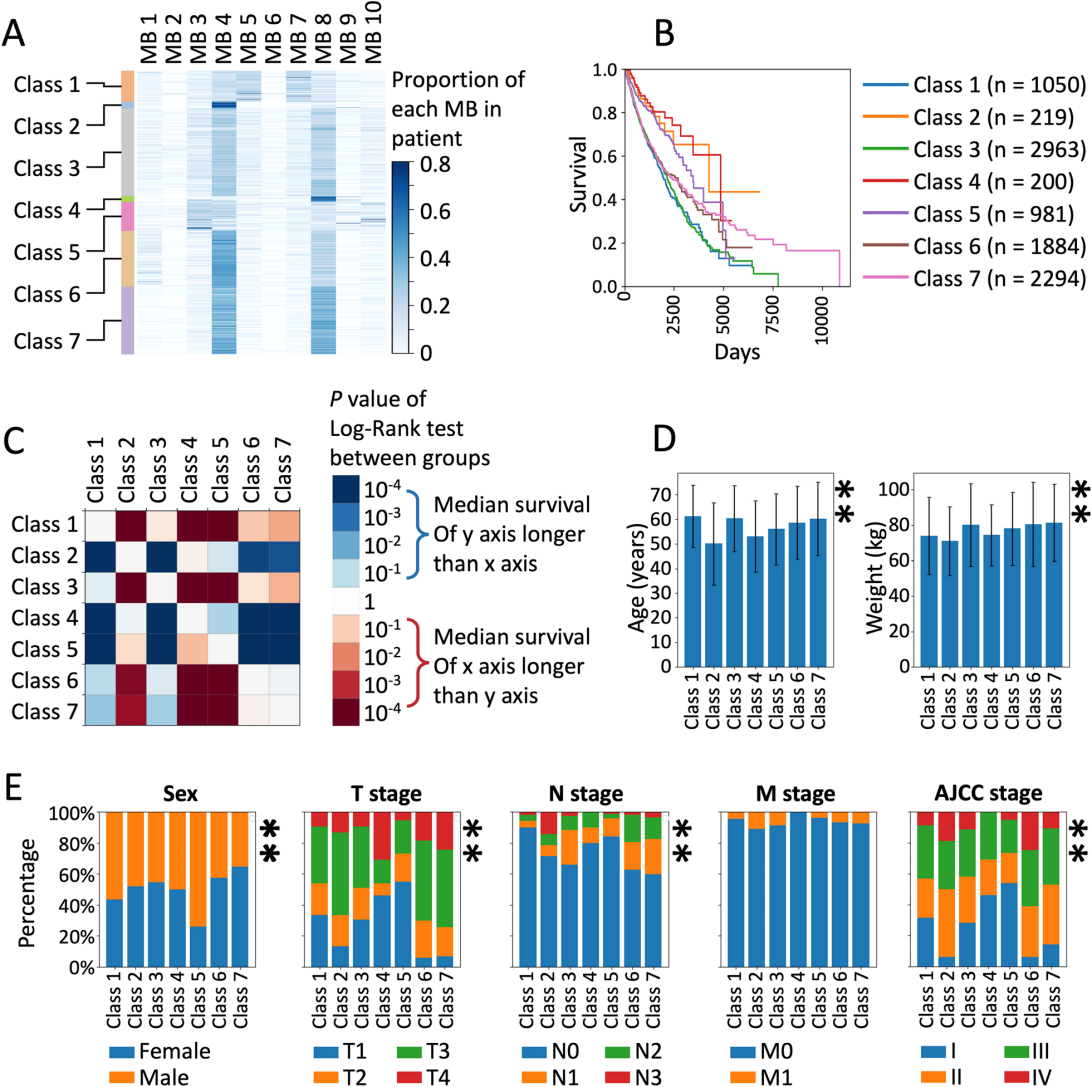
Differences in survival and clinical features between patients clustered according to MB composition. **a** Characteristics of MB composition in patients of 7 classes clustered by the K-means method; each line represents one patient. **b** Survivorship curve of each class of patients. **c** Log-rank test between classes; differences in the p value are reflected in color. **d** e: Clinical features of patients in different classes (*: *p* < 0.05 ANOVA or the chi-square test; **: *p* < 0.005 ANOVA or the chi-square test; error bars represent standard deviation)

To further analyze the influence of the MB composition on the clinical features of patients, a K-means clustering method was used to classify patients according to MB composition. Different kinds of MBs were recorded according to their proportion rather than their number in a single patient. K = 7 was selected as the number of classes to be distinguished. Clustered patients were designated as Classes 1–7. The compositions of MBs in different cancers are shown in Fig. 6A.

In the survival analysis, significant differences in survival curves were observed in different classes of patients (Fig. 6B). In the pairwise survival analysis, patients in Classes 2, 4, and 5 showed better survival, and patients in Classes 1, 3, 6, and 7 showed worse survival (Fig. 6C). In the analysis of specific cancers, survival in different classes of patients generally followed the results obtained for the total sample but with some discrepancies that were not significant. Class 3 patients, in particular, seemed to show poor survival for most of the analyzed cancers (Additional file 1: Figure S14).

Patients of different classes showed distinct clinical features (Fig. 6D, E). According to AJCC staging, a significantly lower proportion of stage IV patients and a higher proportion of stage I patients were observed in Classes 4 and 5, which may be related to the better survival of these 2 classes of patients. Interestingly, Class 4 included significantly more T4 patients but hardly any M1 patients. This suggests that the MB composition of Class 4 may be associated with the local progression of cancers. Class 6 patients showed the highest percentage of AJCC stage 4 and lowest percentage of AJCC stage I, which may be the reason for the poor survival of these patients. In the analysis of age, patients of Class 3 were found to present significantly greater ages. At the same time, the weight of Class 3 patients was also high. Class 1 patients exhibited a high percentage of AJCC stage 1 and a low percentage of stage IV. Moreover, the proportion of N0 patients in Class 1 was significantly higher than that in other classes.

## Discussion

Several studies on mutation signatures have been published. Most of the studies were based on the TCGA or other databases. Several mathematical methods are now used to cluster the mutation signature [2, 4, 9, 11, 12]. Some of the studies have suggested that adjacent bases may affect the characteristics of the mutation signature.

An increase in the number of included flanking bases leads to an exponential increase in the number of possible classifications. In our study, together with the 50 flanking bases on both sides, there were theoretically 6 × 4^100^ possible classes, making it nearly impossible to analyze such classes with classical statistical methods. LSTM is a machine learning approach that is good at extracting the features of long sequences [32]. This approach provided us with a method for extracting the features of mutated sequences across a wider spatial scope. A follow-up SOM method can then be used to discover internal relationships between the extracted features and ultimately obtain different categories of mutant sequences. To avoid overfitting of the model, the weight of the vectors in the competitive layer was updated after all input data were trained in one batch. Each iteration of training included 2 LSTM iterations and 2 SOM iterations. In this way, we identified 10 classes of mutation sequences. No one kind of mutation was contained in a single class of MBs. The composition of the bases flanking the mutation sites differed considerably. Generally, units located far from the endpoint had less influence on the LSTM output than those located close to the endpoint [26]. This characteristic was reflected in the flanking bases of the mutation site. In all kinds of MBs, the proportions of A, T, C, and G were quite different among the bases near the mutation site. With an increase in the distance from the mutation site, the proportions of the four bases tended to become balanced.

The analysis of MBs in different kind of cancers suggested that MBs may comprehensively reflect the difference in cancers according to both location and pathological type. Previous studies have proven that different mutation signatures may be associated with different triggers involved in various mutation processes and result in differing biological behaviors of cancers [2, 9]. A variety of mutation signatures that may be related to the biology and etiology of cancer have been identified [2, 9, 14–16, 33–36]. Our study suggests that a high incidence of MB 4 and MB 8 is associated with pathologic types of cancer that are believed to be caused by external mutagenic exposure, such as SCC, transitional cell carcinoma, malignant mesothelioma, and complex epithelial carcinoma. We also found that some kinds of cancer, such as melanoma and transitional cell carcinoma, had distinctive features that are worthy of further study to determine the relationship between each MB and specific cancer processes. In a given gene, SBSs may occur at different bases with different features and present as different kinds of MBs. Each gene with a high mutation frequency contained multiple kinds of MBs. On further study of the MB proportion in genes that are highly frequently mutated, we observed differences in the mutated gene compositions of different MBs. This finding suggests that attention should be paid to the effect of different MBs on the characteristics of cancer when they occur in the same gene.

Then, in the subsequent analysis, we focused on the relationship between MBs and clinical features, including survival. First, survival analysis between patients with different MBs in the same gene showed a significant correlation between survival and MBs for specific genes. In the analysis between MBs and clinical features, it was observed that the proportion of MBs generally showed an obvious tendency with a change in clinical features, which suggests that characteristics of MBs reflect the characteristics of cancers. Considering the differences in the clinical significance of staging in different cancers, further analysis was performed on each cancer with high incidence. Generally, in most cancers, the MB composition in patients with different clinical features basically followed the pattern observed in all samples. While there were some exceptions, such as in stomach cancer and colon cancer, MB tendencies in T and N stages were opposite to those in the entire sample and to other cancers with high incidence. This result suggests that local and lymph node progression in gastrointestinal cancers may exhibit distinct mechanisms. In the analysis of age, younger and older patients showed similar MB compositions in the form of a conic structure in the bar graph. This suggests that the similar cancer biologies of young and old patients require further study. Generally, although the results showed a clear relationship between MBs and clinical features, details of the relationship as well as its mechanism still require further study.

To further explore the translational relevance of MBs, we then clustered patients into 7 classes according to MB composition. Interestingly, patients with a balanced composition of MBs (Classes 1, 3 and 5, especially Class 3) were associated with poor survival for most of the analyzed cancers. These results suggest that a balanced MB composition may predict poor survival in patients and may be related to mixed mutation triggers. Some classes of patients showed typical clinical features. For example, patients in Class 3 were older and weighed more than those in the other classes. These factors may be partly responsible for the poor survival of patients in Class 3. In contrast, although the patients in Class 1 were older, they did not weigh more than those in the other classes. Therefore, further study is still needed to determine the mechanism by which patients in Class 1 experience poor survival. Due to the natural differences in cancer incidence, large differences exist between different cancers. In different cancers, MB may be involved in different kinds of cancer-related processes. Therefore, the analysis of the relationship between MBs and distinctive clinical features in specific kinds of cancer can provide more information about how MBs are related to cancer etiology, processes, prognosis and drug susceptibility.

There were still some constraints and limitations to this study. The clustering results obtained from the LSTM-SOM model were largely dependent on the selection of SOM parameters (especially the neighborhood function parameter). There exists the possibility that when training with other parameters, the classification obtained may have been related to clinical features that were not included in this study and thus need further study. Moreover, the mechanism of machine learning models is difficult to explain [37]. It would be meaningful to use a mathematical method to explore the mechanism of the LSTM-SOM functions to improve the interpretability of the LSTM-SOM model and to explain the formation of different classes of MB to determine how sequences of bases affect the characteristics of cancers. Different MBs may also be involved in complex changes in three-dimensional chromosome conformation. Moreover, molecular biology methods are helpful for explaining the different characteristics of MBs.

## Conclusion

This study provided a method for analyzing the characteristics of mutant sequences. Result of this study showed that flanking sequences, together with mutation bases, shape the signatures of SBSs. The analysis of MBs in different kind of cancers suggested that MBs reflect the difference in cancers according to both location and pathological type. Mutation sequence signatures (MBs) identified via LSTM-SOM method in this study were shown related to clinical features and survival of cancer patients. Composition of MBs is a feasible predictive factor of clinical prognosis. Patients with balanced MB composition seems to have worse survival. Further study on the interpretability of LSTM-SOM network and on the mechanism of MBs related to cancer characteristics is suggested.

## Supplementary Information

**Additional file 1.** Supplementary Figures.

**Additional file 2.** Supplementary Tables.

## Data Availability

SBS data and clinical data of patients enrolled in this study were obtained from the TCGA database. The.maf files including SBS somatic mutation data were downloaded from https://portal.gdc.cancer.gov/repository/https://portal.gdc.cancer.gov/repository?filters=%7B%22op%22%3A%22and%22%2C%22content%22%3A%5B%7B%22op%22%3A%22in%22%2C%22content%22%3A%7B%22field%22%3A%22files.access%22%2C%22value%22%3A%5B%22open%22%5D%7D%7D%2C%7B%22op%22%3A%22in%22%2C%22content%22%3A%7B%22field%22%3A%22files.data_category%22%2C%22value%22%3A%5B%22simple%20nucleotide%20variation%22%5D%7D%7D%5D%7D. The.xml files including clinical data were downloaded from https://portal.gdc.cancer.gov/repository?filters=%7B%22op%22%3A%22and%22%2C%22content%22%3A%5B%7B%22op%22%3A%22in%22%2C%22content%22%3A%7B%22field%22%3A%22files.data_category%22%2C%22value%22%3A%5B%22clinical%22%5D%7D%7D%5D%7D. Reference genome sequences (Genome Reference Consortium human genome build 38, GRCh38) were downloaded from https://hgdownload.soe.ucsc.edu/goldenPath/hg38/bigZips/latest/hg38.fa.gz).

## Abbreviations

LSTM: Long short-term memory
SOM: Self-organizing map
SBS: Single-base substitution
MB: Mutation blot
TCGA: The Cancer Genome Atlas
GRCh38: Genome reference consortium human genome build 38
AJCC: American Joint Committee on Cancer
AC: Adenocarcinoma
SCC: Squamous cell carcinoma
