# Supplementary material for: Clinical feature-related single-base substitution sequence signatures identified with an unsupervised machine learning approach
Source: BMC Med Genomics. 2021 Dec 20;14:298. doi: 10.1186/s12920-021-01144-1 (PMC8686331; doi:10.1186/s12920-021-01144-1)
Supplement: Supplementary file 1 — Additional file 1. Supplementary Figures. [file 12920_2021_1144_MOESM1_ESM.docx]

**
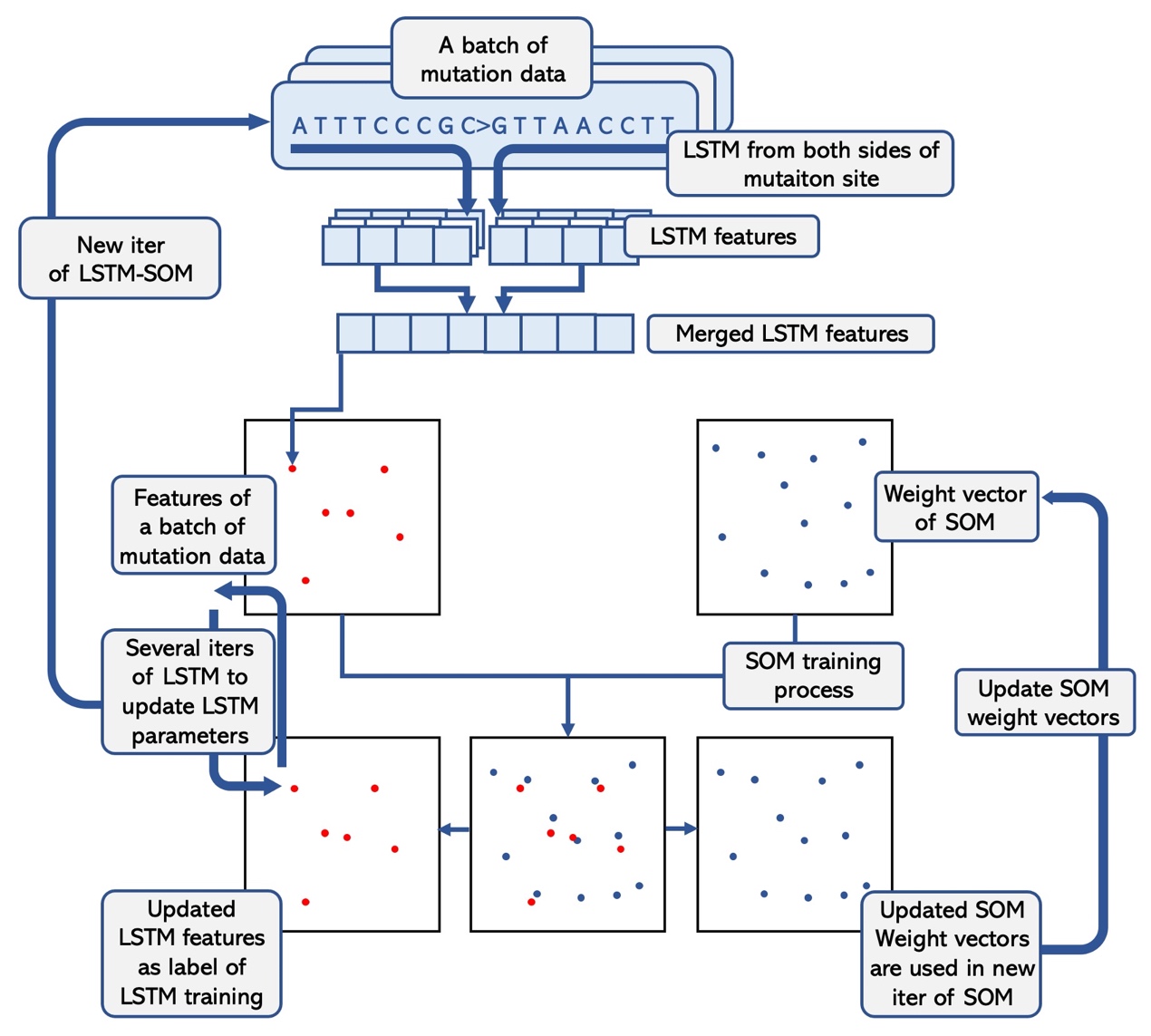
**

**Figure S1. Flowchart of LSTM-SOM model.**

**
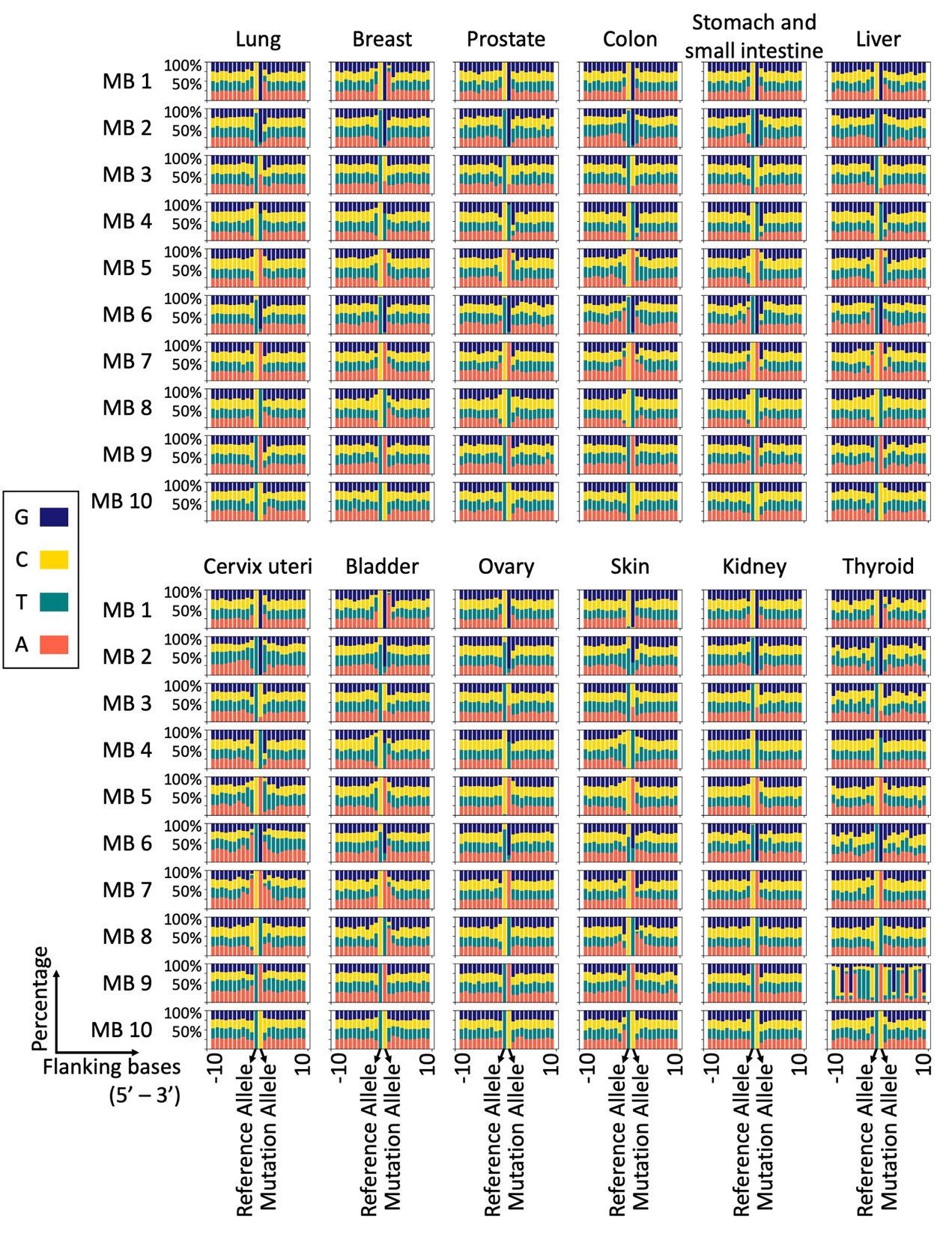
**

**Figure S2. Mutation type and composition of flanking bases of each MB in cancers with high incidence.**


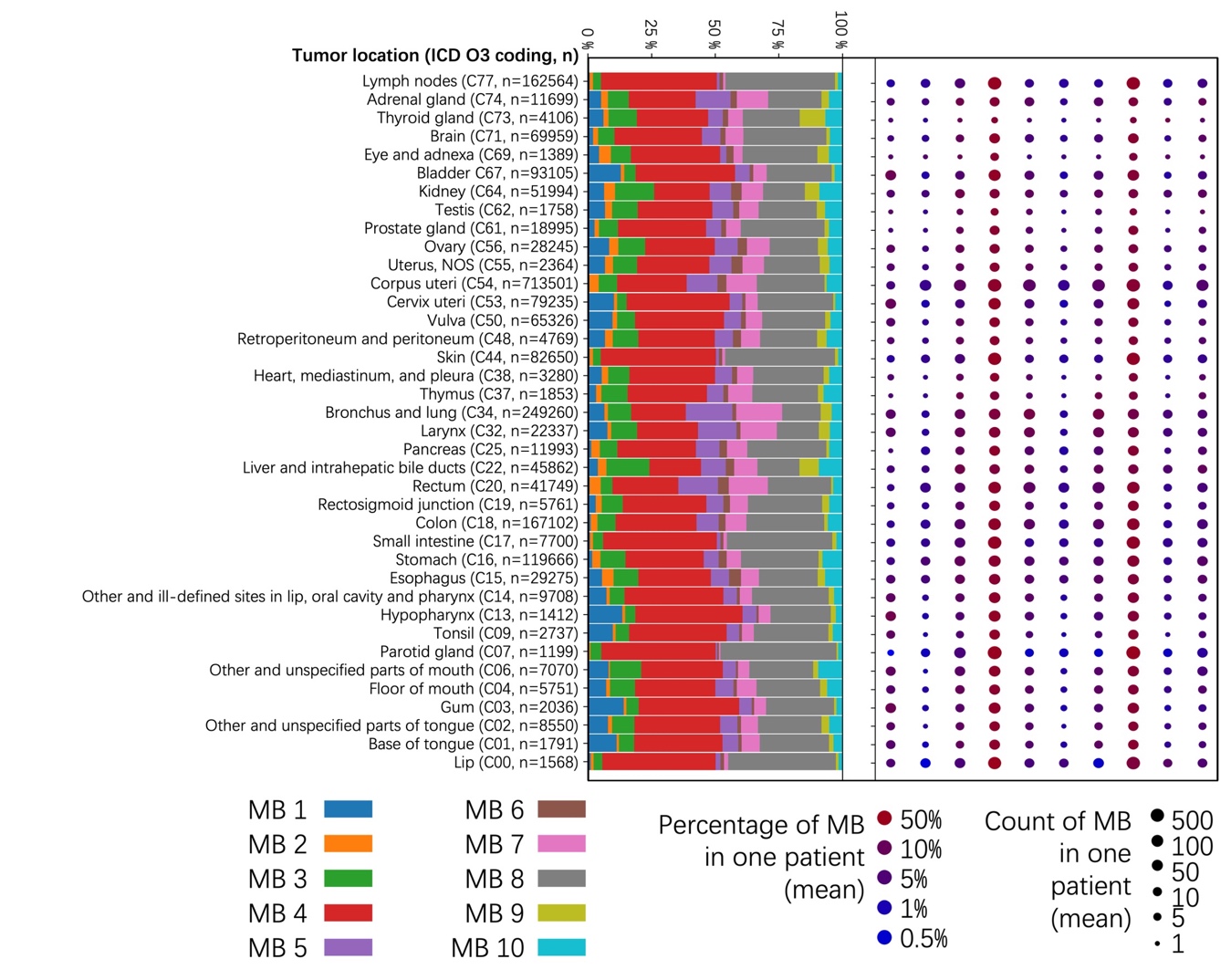


**Figure S3. Quantity and proportion of MBs in different cancers according to ICD O3 site code.** The left subgraph shows the proportion of different MBs in all SBS mutation data points from different kinds of cancers. The right subgraph shows the quantity and proportion of different MBs in patients. Differences in quantity are reflected in the size of the point, and differences in proportion are reflected in the color of the point.

**
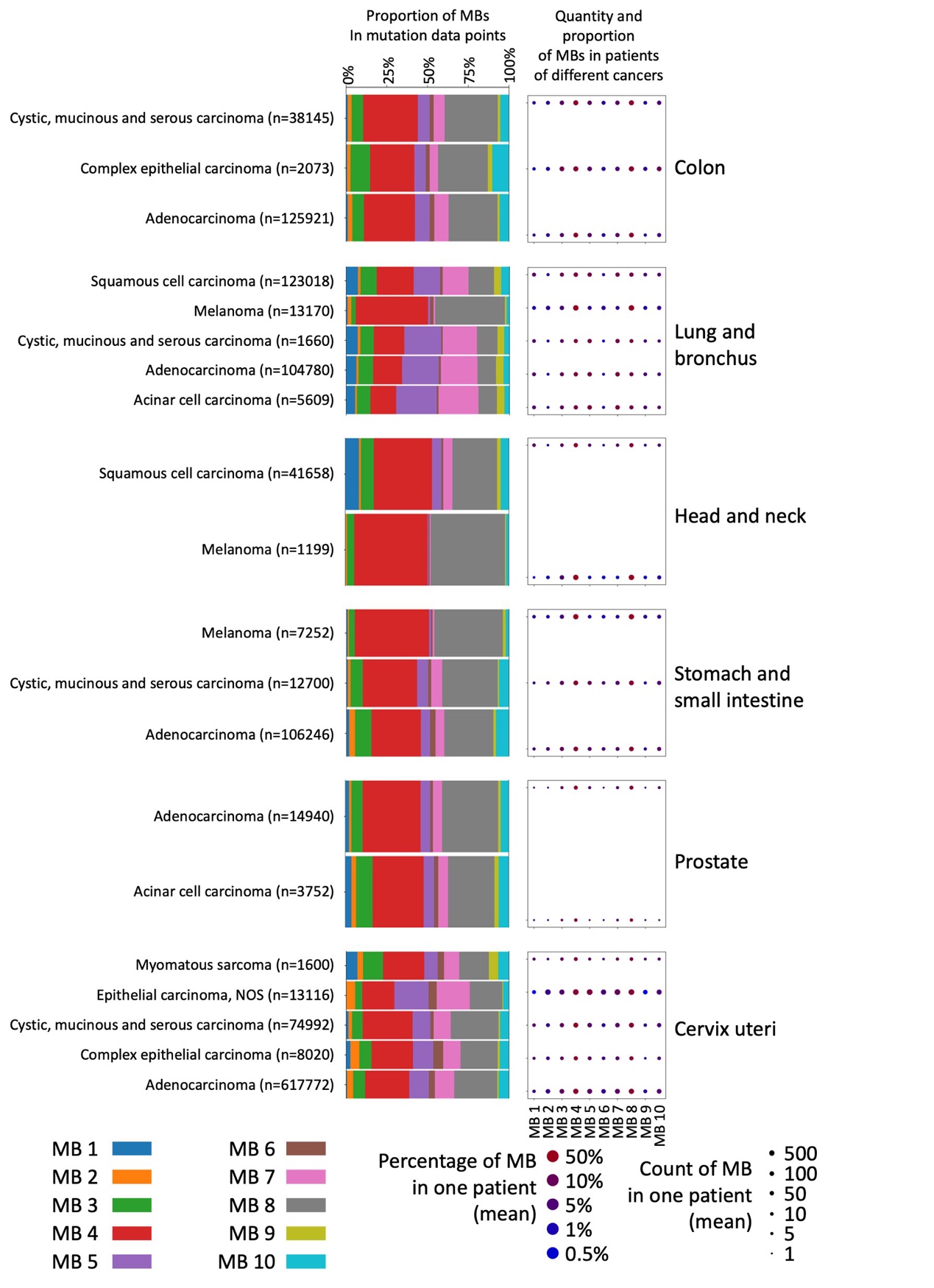
**

**Figure S4. Quantity and proportion of MBs in cancers with high incidence and multiple types of pathology.** For each cancer, the left subgraph shows the proportion of different MBs in SBS mutation data points, and the right subgraph shows the quantity and proportion of different MBs in patients. Differences in quantity are reflected in the size of the point, and differences in proportion are reflected in the color of the point.


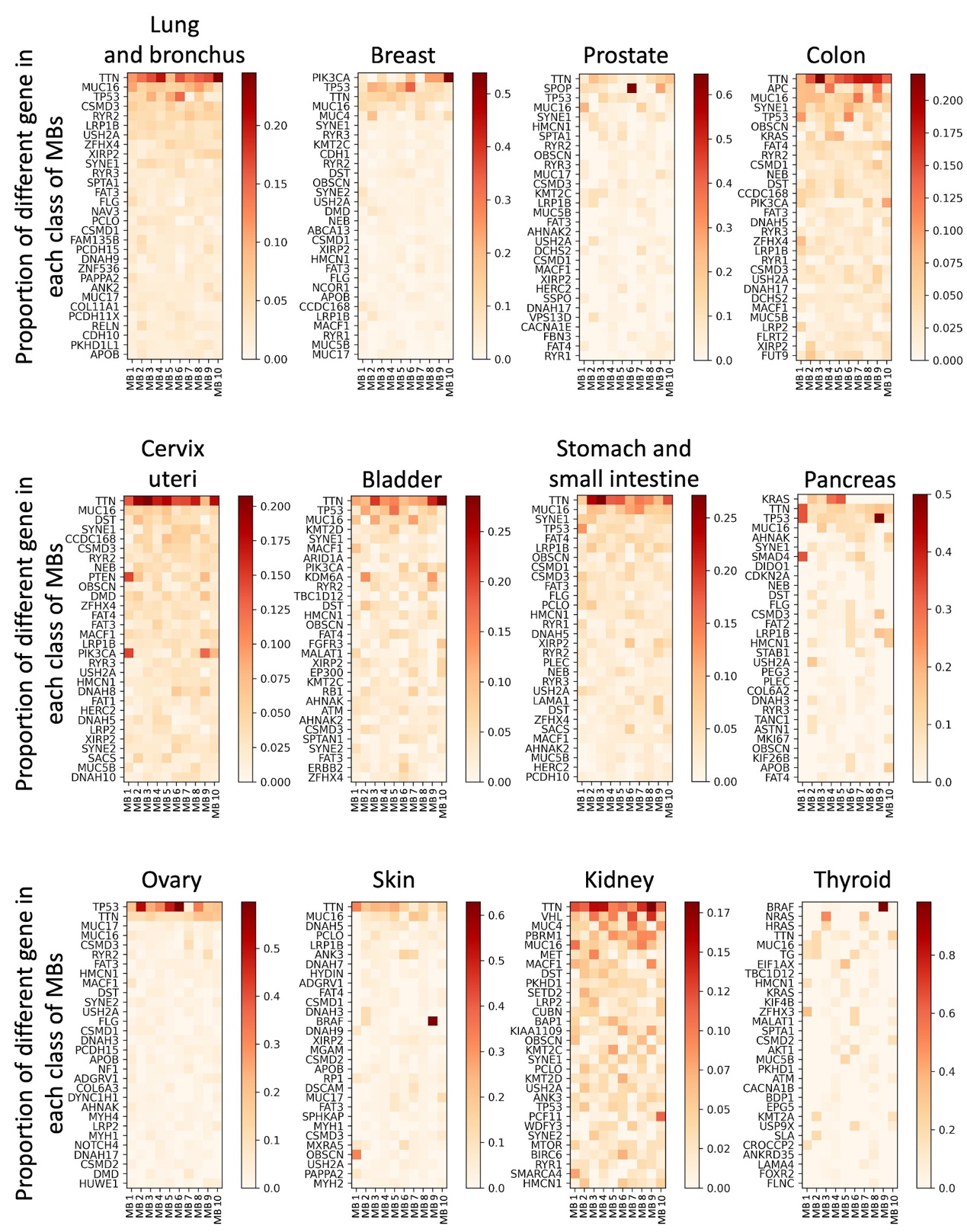


**Figure S5. Genes with high mutation frequency in different MBs in cancers with high incidence.**

**
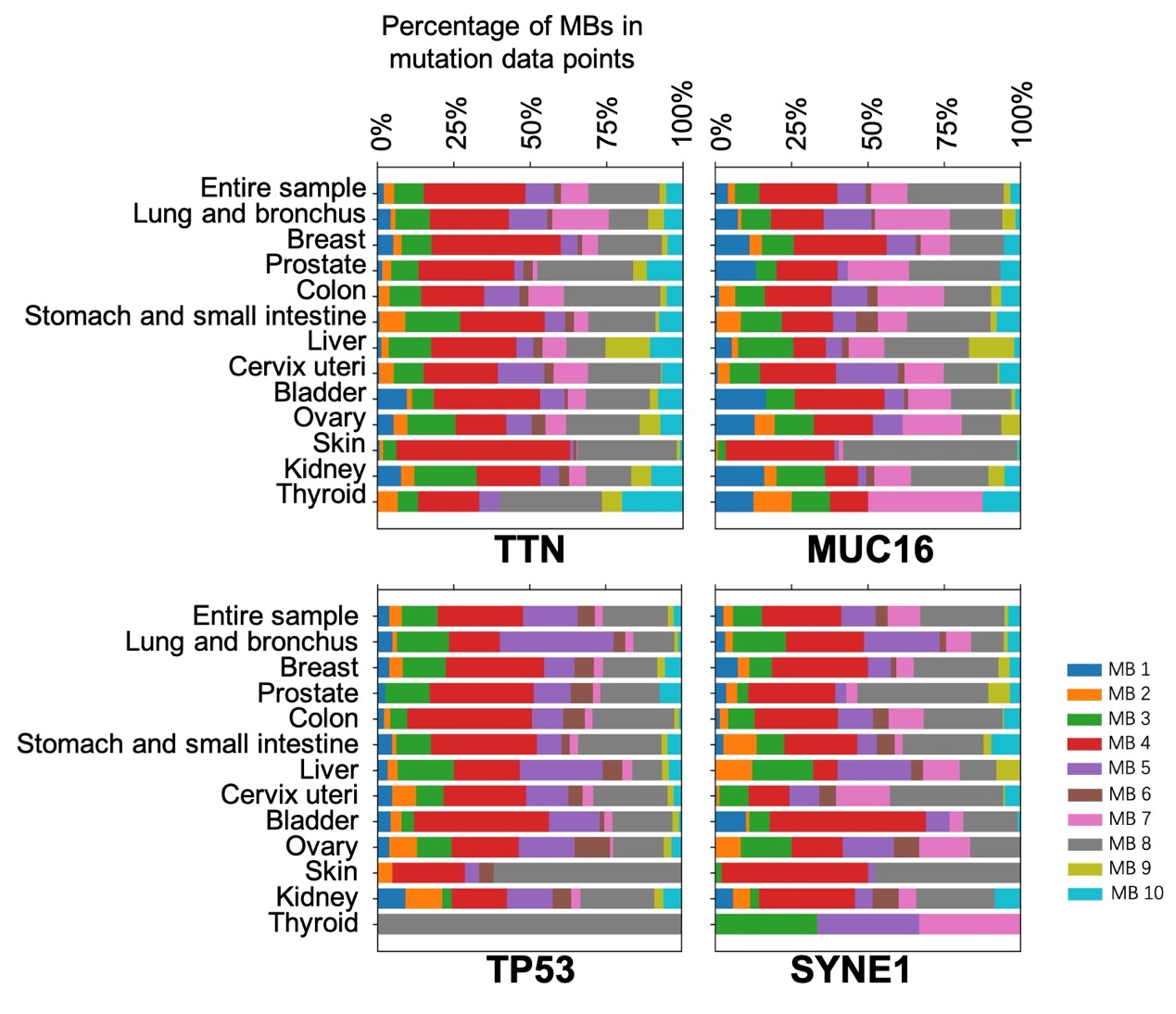
**

**Figure S6. Proportion of MBs in genes with high mutation frequencies from different cancers**

**
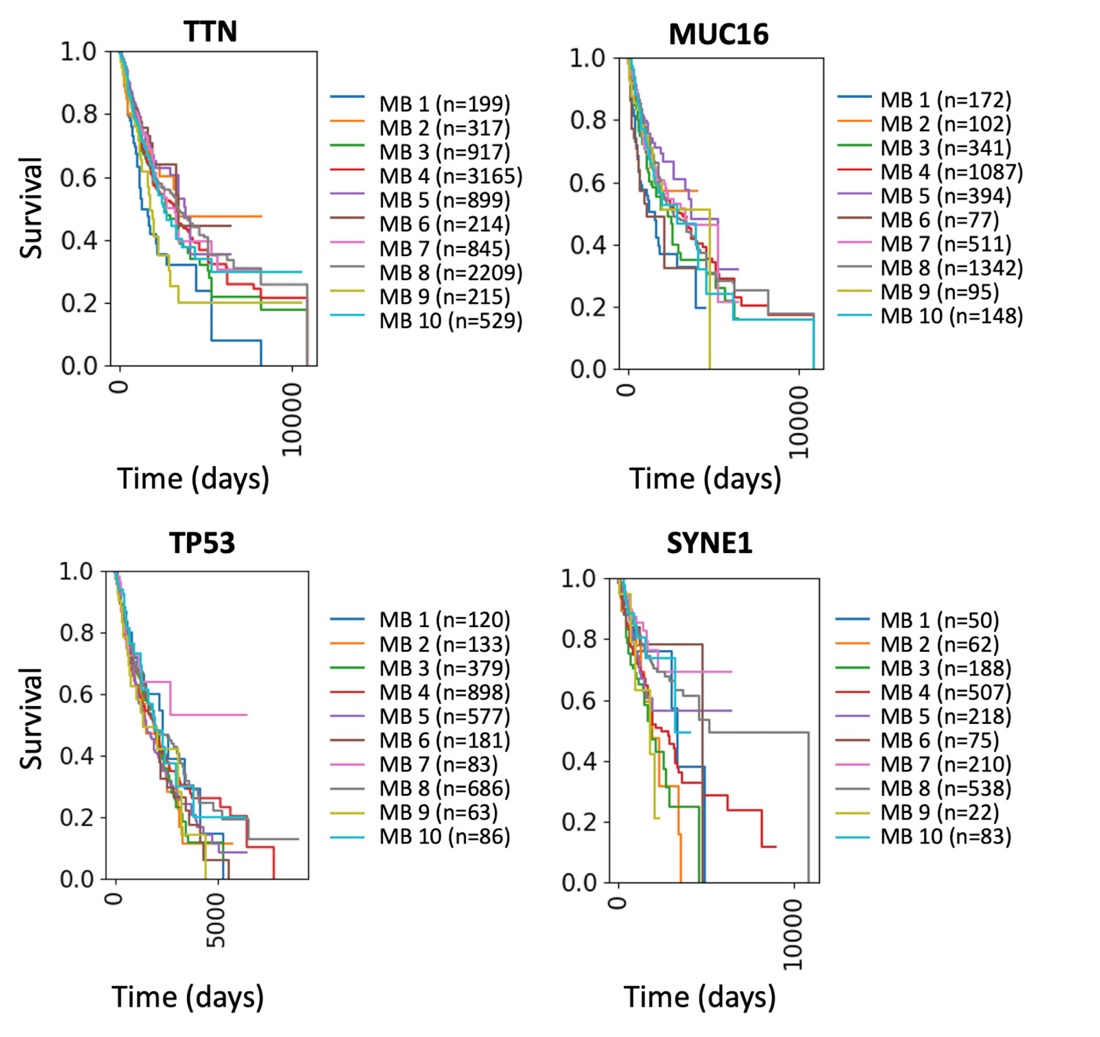
**

**Figure S7. Survivorship curve of patients with different MBs in TTN, MUC16, TP53, and SYNE1.**

**
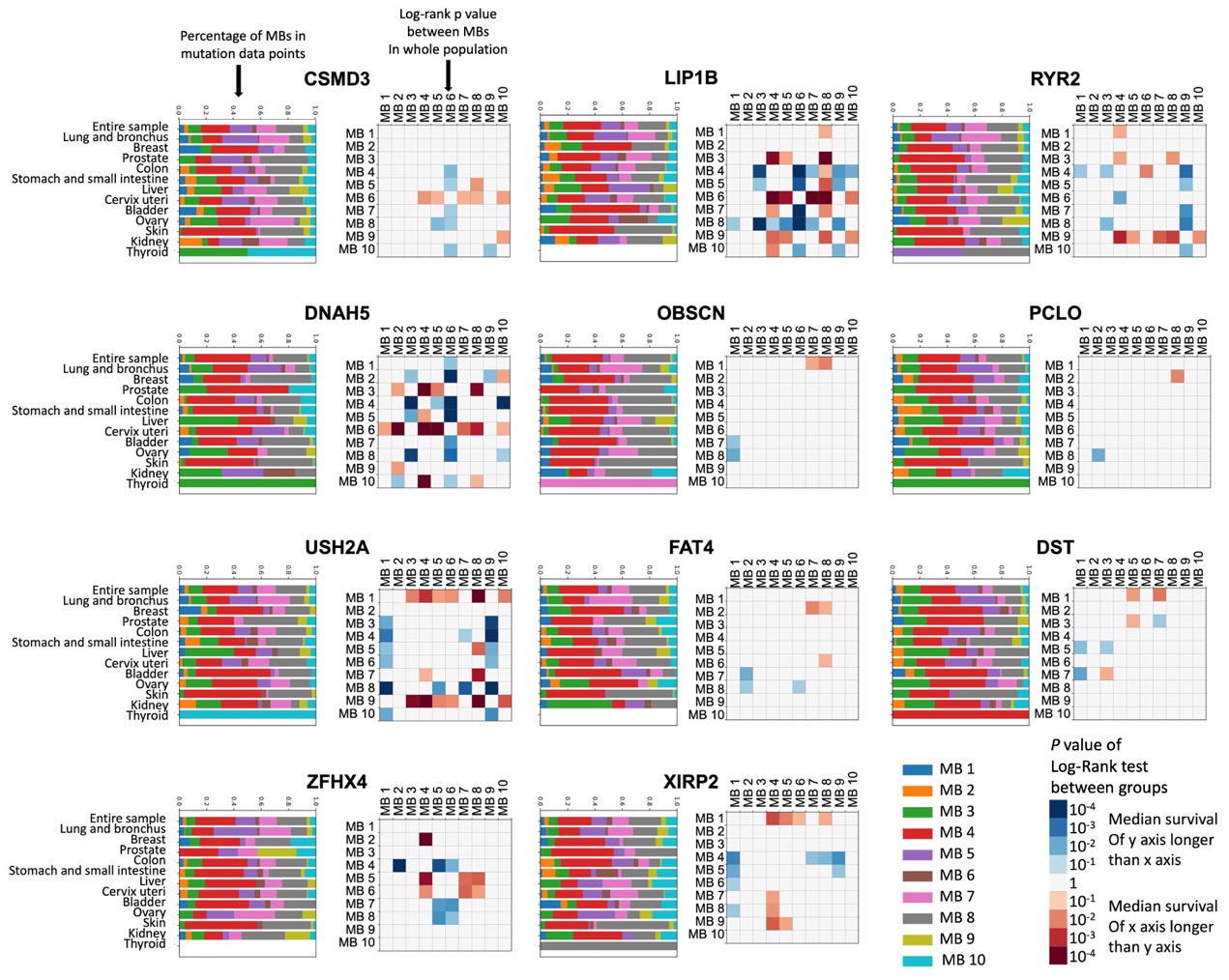
**

**Figure S8. Relationship between patient survival and MB in genes with high mutation frequencies.** Genes ranked 5-15 in mutation frequency are shown. For each gene, the left subgraph shows the proportion of MB in all mutation data points from different cancers; and the right subgraph shows the *P* value of the log-rank test between groups in the whole population. Only *P* values less than 0.05 are shown in the heat map.


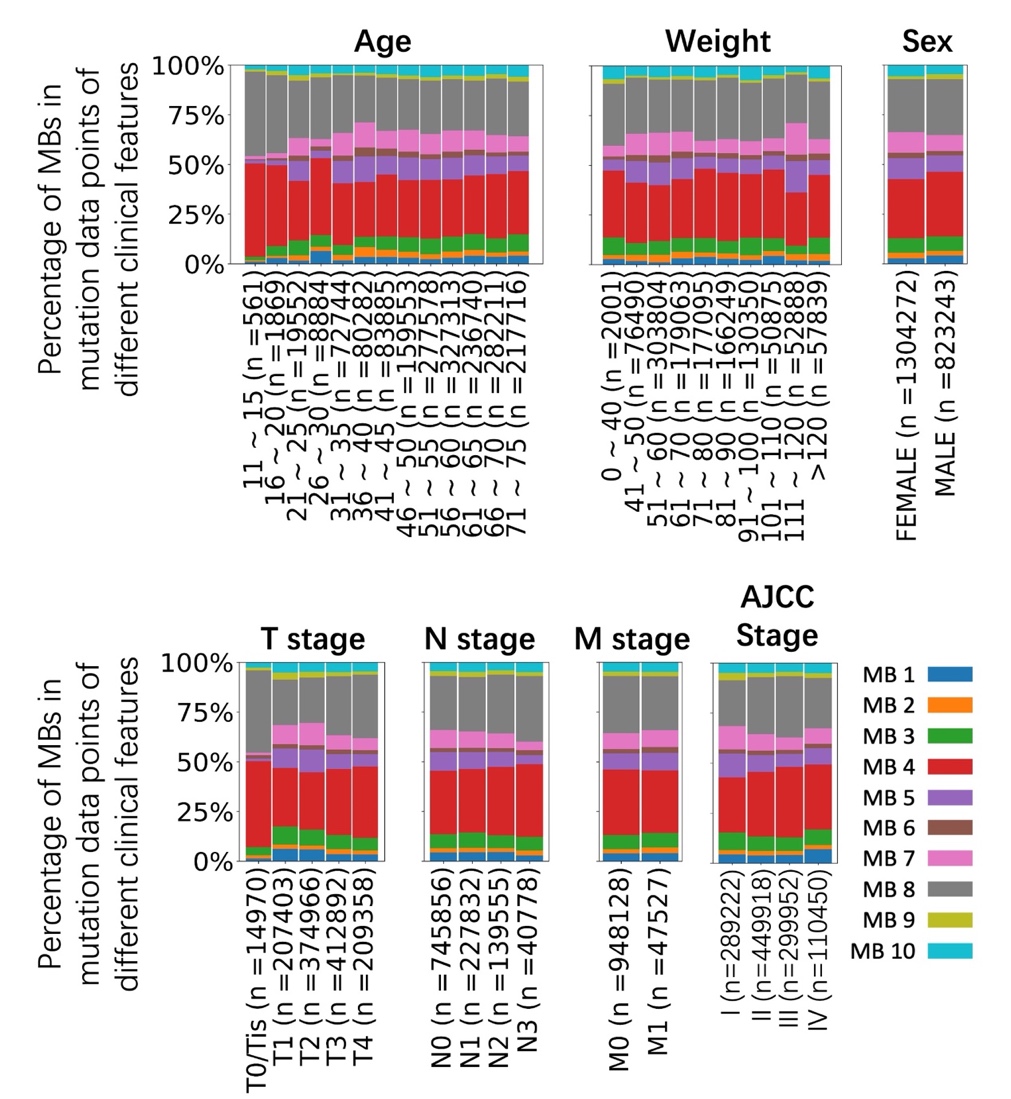


**Figure S9. Proportion of MB in patients of different clinical features.**


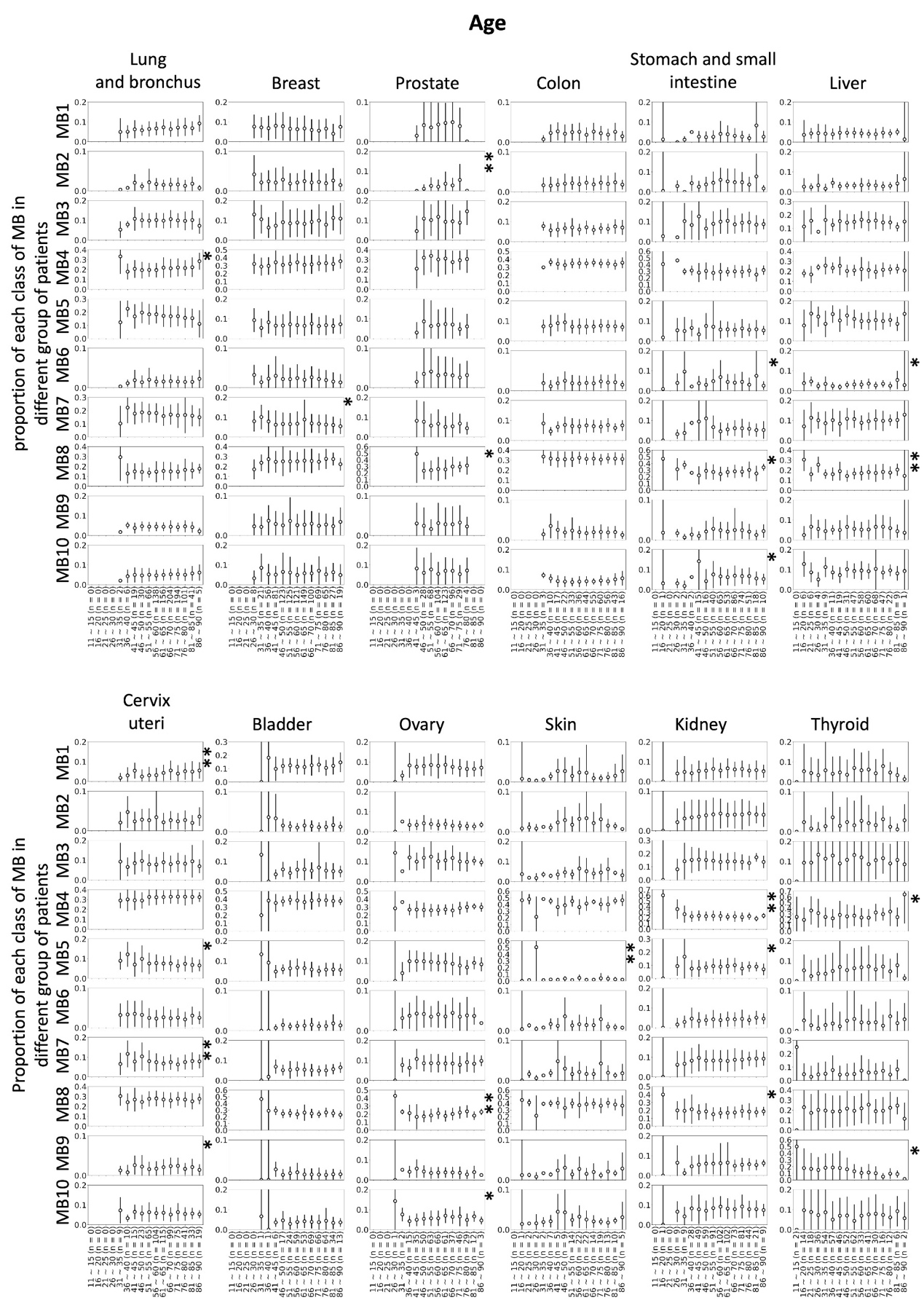


**Figure S10. Statistics of the proportion of each MB by age in cancers with high incidence.** *: *P < 0.05* in the t test or ANOVA between groups; ** *P < 0.005* in the t test or ANOVA between groups.

**
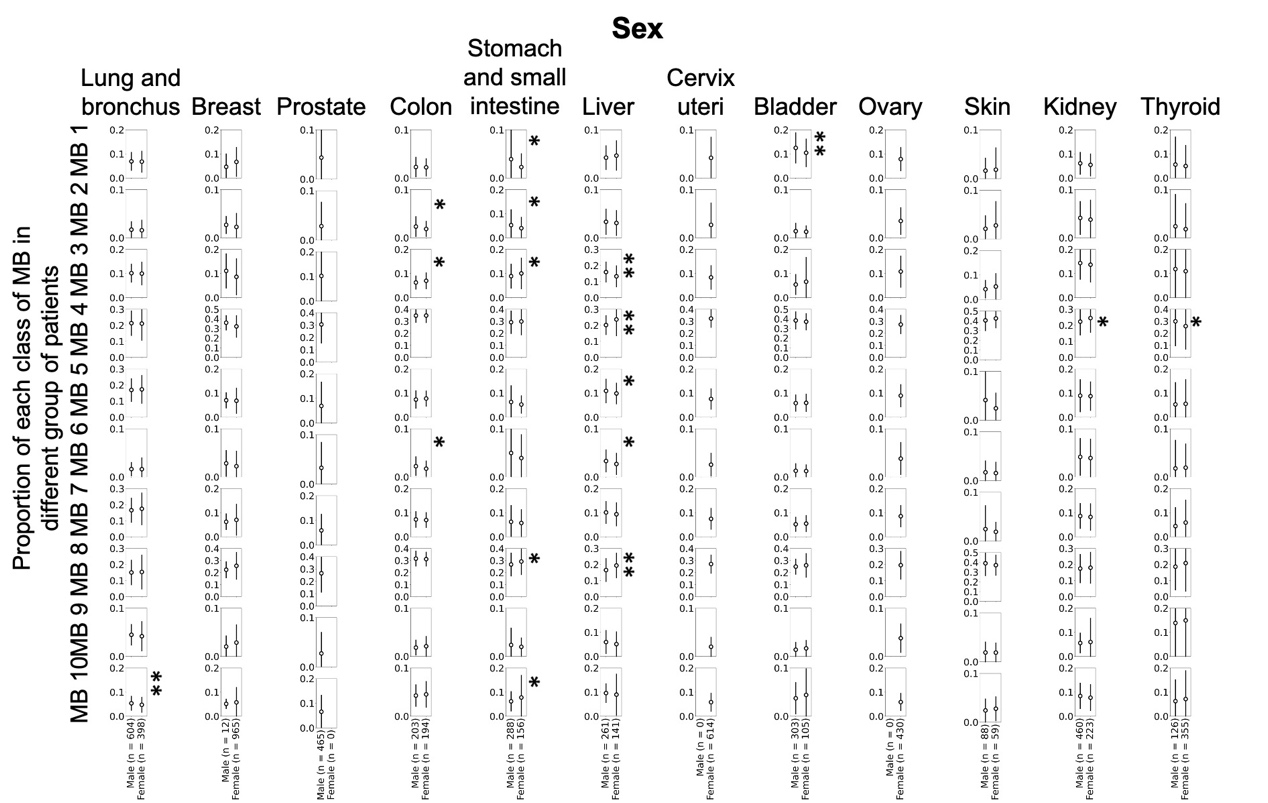
**

**Figure S11. Statistics of the proportion of each MB by sex in cancers with high incidence.** *: *P < 0.05* in the t test or ANOVA between groups; ** *P < 0.005* in the t test or ANOVA between groups.

**
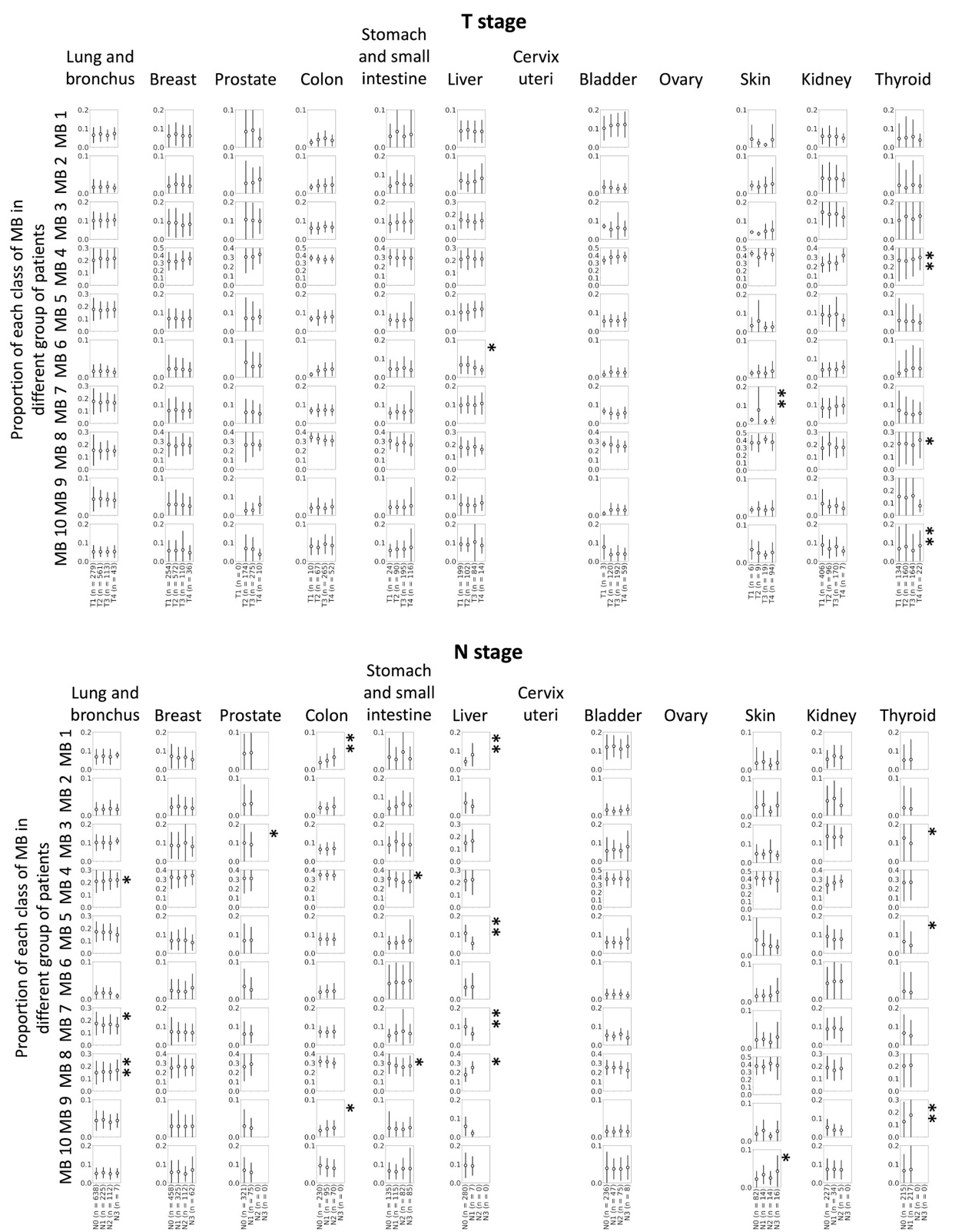
**

**Figure S12. Statistics of the proportion of each MB by T stage and N stage in cancers with high incidence.** *: *P < 0.05* in the t test or ANOVA between groups; ** *P < 0.005* in the t test or ANOVA between groups.

**
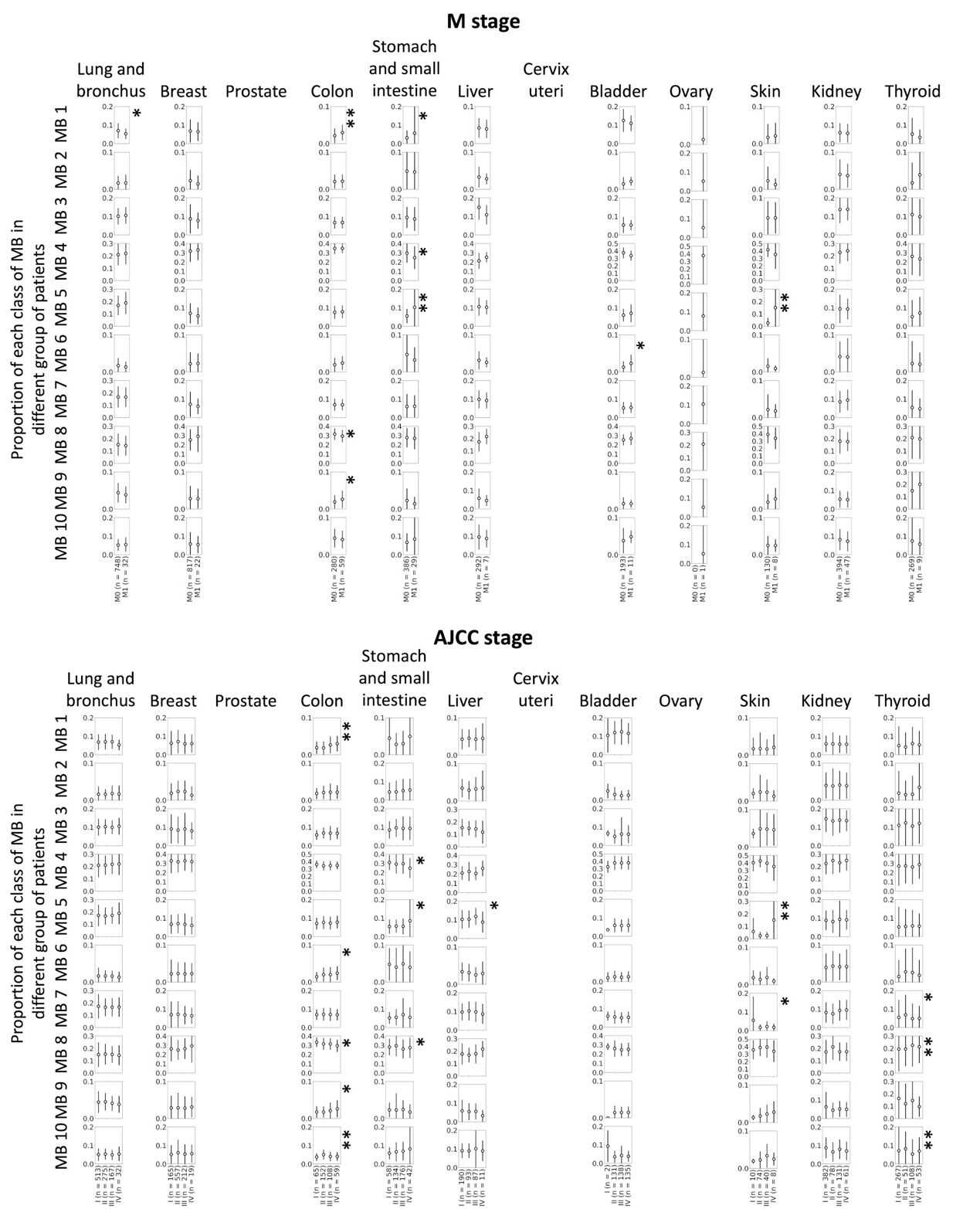
**

**Figure S13. Statistics of the proportion of each MB by M stage and AJCC stage in cancers with high incidence.** *: *P < 0.05* in the t test or ANOVA between groups; ** *P < 0.005* in the t test or ANOVA between groups.


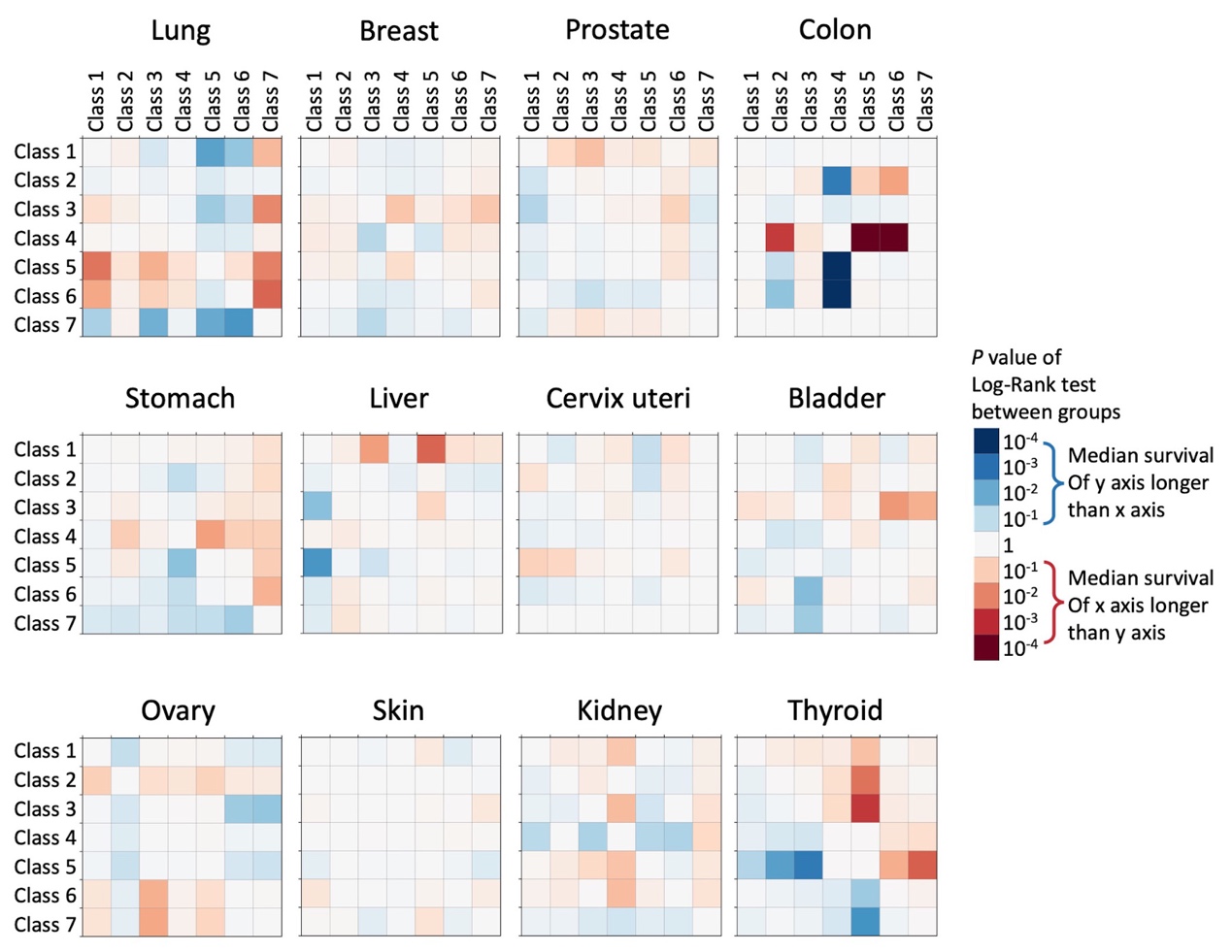


**Figure S14. Log-rank test between different classes of patients in different cancers.** Differences in the *P* value are reflected in color.
