# Supplementary material for: Clinical feature-related single-base substitution sequence signatures identified with an unsupervised machine learning approach
Source: BMC Med Genomics. 2021 Dec 20;14:298. doi: 10.1186/s12920-021-01144-1 (PMC8686331; doi:10.1186/s12920-021-01144-1)
Supplement: Supplementary file 2 — Additional file 2. Supplementary Tables. [file 12920_2021_1144_MOESM2_ESM.docx]

**Table S1. Count of mutation type in each class of MB.**

|  | Mutation type | | | | | |
| --- | --- | --- | --- | --- | --- | --- |
|  | **C>A** | **C>G** | **C>T** | **T>A** | **T>C** | **T>G** |
| MB 1 | 0 | 68877 | 0 | 0 | 346 | 152 |
| MB 2 | 559 | 0 | 2595 | 239 | 0 | 53892 |
| MB 3 | 0 | 0 | 0 | 41995 | 114665 | 0 |
| MB 4 | 0 | 70276 | 587893 | 0 | 0 | 0 |
| MB 5 | 205678 | 313 | 0 | 0 | 403 | 126 |
| MB 6 | 552 | 1 | 2645 | 210 | 0 | 52238 |
| MB 7 | 201605 | 0 | 0 | 0 | 0 | 0 |
| MB 8 | 0 | 0 | 583264 | 0 | 0 | 0 |
| MB 9 | 0 | 0 | 0 | 40582 | 0 | 0 |
| MB 10 | 0 | 0 | 0 | 0 | 112421 | 0 |

**Table S2. Genes with high frequency in different classes of MBs** (Entire sample, genes with top 15 mutation frequency are shown).

| **MB 1** | | | **MB 2** | | | **MB 3** | | | **MB 4** | | | **MB 5** | |
| --- | --- | --- | --- | --- | --- | --- | --- | --- | --- | --- | --- | --- | --- |
| Gene | Frequency | Gene | | Frequency | Gene | | Frequency | Gene | | Frequency | Gene | | Frequency |
| TTN | 0.287% | TTN | | 0.553% | TTN | | 0.585% | TTN | | 0.481% | TTN | | 0.430% |
| MUC16 | 0.248% | TP53 | | 0.232% | TP53 | | 0.242% | MUC16 | | 0.165% | TP53 | | 0.279% |
| TP53 | 0.173% | MUC16 | | 0.178% | MUC16 | | 0.218% | TP53 | | 0.137% | MUC16 | | 0.191% |
| MACF1 | 0.110% | DST | | 0.138% | LRP1B | | 0.122% | DNAH5 | | 0.100% | CSMD3 | | 0.147% |
| CSMD3 | 0.094% | PCLO | | 0.133% | SYNE1 | | 0.120% | OBSCN | | 0.089% | LRP1B | | 0.126% |
| FLG | 0.092% | DMD | | 0.122% | DST | | 0.113% | SYNE1 | | 0.077% | DST | | 0.117% |
| SYNE2 | 0.089% | LRP1B | | 0.120% | CSMD3 | | 0.110% | LRP1B | | 0.076% | KRAS | | 0.115% |
| RYR2 | 0.086% | PTEN | | 0.108% | PCLO | | 0.106% | FAT3 | | 0.073% | SYNE1 | | 0.106% |
| USH2A | 0.078% | SYNE1 | | 0.108% | USH2A | | 0.097% | RYR2 | | 0.070% | RYR2 | | 0.099% |
| OBSCN | 0.075% | CSMD3 | | 0.096% | RYR2 | | 0.096% | PCLO | | 0.070% | ZFHX4 | | 0.099% |
| SYNE1 | 0.072% | SACS | | 0.094% | FAT4 | | 0.083% | FAT4 | | 0.064% | CCDC168 | | 0.091% |
| PIK3CA | 0.072% | NEB | | 0.091% | NEB | | 0.082% | RYR1 | | 0.064% | DNAH5 | | 0.090% |
| FAT1 | 0.071% | TSPAN15 | | 0.086% | DMD | | 0.073% | ZFHX4 | | 0.064% | NEB | | 0.088% |
| LRP1B | 0.069% | XIRP2 | | 0.082% | NRAS | | 0.072% | NEB | | 0.063% | DMD | | 0.082% |
| ADGRV1 | 0.063% | ADGRG4 | | 0.080% | DNAH5 | | 0.070% | FLG | | 0.061% | MUC17 | | 0.081% |

| **MB 6** | | **MB 7** | | **MB 8** | | **MB 9** | | **MB 10** | |
| --- | --- | --- | --- | --- | --- | --- | --- | --- | --- |
| Gene | Frequency | Gene | Frequency | Gene | Frequency | Gene | Frequency | Gene | Frequency |
| TTN | 0.385% | TTN | 0.420% | TTN | 0.379% | BRAF | 1.264% | TTN | 0.471% |
| TP53 | 0.325% | MUC16 | 0.253% | MUC16 | 0.230% | TTN | 0.530% | PIK3CA | 0.263% |
| MUC16 | 0.138% | RYR2 | 0.146% | TP53 | 0.118% | MUC16 | 0.234% | MUC16 | 0.132% |
| SYNE1 | 0.135% | CSMD3 | 0.133% | PIK3CA | 0.101% | CSMD3 | 0.165% | XIRP2 | 0.102% |
| XIRP2 | 0.102% | SYNE1 | 0.104% | SYNE1 | 0.092% | TP53 | 0.155% | CSMD3 | 0.095% |
| CSMD3 | 0.101% | USH2A | 0.101% | OBSCN | 0.091% | RYR2 | 0.145% | LRP1B | 0.093% |
| ABCA13 | 0.097% | FAT4 | 0.094% | CSMD1 | 0.074% | PIK3CA | 0.133% | MACF1 | 0.092% |
| DNAH8 | 0.090% | LRP1B | 0.094% | RYR2 | 0.071% | MYBBP1A | 0.131% | MUC4 | 0.091% |
| USH2A | 0.084% | ZFHX4 | 0.090% | DNAH5 | 0.069% | XIRP2 | 0.126% | RYR2 | 0.082% |
| LRP1B | 0.084% | APOB | 0.087% | FAT4 | 0.067% | LRP1B | 0.111% | SYNE2 | 0.079% |
| FAT4 | 0.084% | XIRP2 | 0.083% | PCLO | 0.067% | PCDH15 | 0.106% | FAT4 | 0.076% |
| BIRC6 | 0.083% | ABCA13 | 0.082% | DNAH17 | 0.067% | ABCA13 | 0.099% | TP53 | 0.076% |
| SYNE2 | 0.081% | RYR3 | 0.079% | DNAH8 | 0.066% | ZFHX4 | 0.091% | ADGRV1 | 0.075% |
| DST | 0.079% | FLG | 0.078% | RYR1 | 0.064% | MACF1 | 0.091% | SYNE1 | 0.074% |
| UNC13C | 0.077% | OBSCN | 0.078% | MUC5B | 0.063% | FAT3 | 0.089% | DNAH5 | 0.073% |
